# Infection kinetics of Covid-19 and containment strategy

**DOI:** 10.1038/s41598-021-90698-2

**Published:** 2021-06-02

**Authors:** Amit K Chattopadhyay, Debajyoti Choudhury, Goutam Ghosh, Bidisha Kundu, Sujit Kumar Nath

**Affiliations:** 1 Mathematics, College of Engineering and Physical Sciences, Aston University, Birmingham, B4 7ET UK; 2 Department of Physics and Astrophysics, University of Delhi, Delhi, 110007 India; 3 Gandhi Institute of Engineering and Technology University, Gunupur, Odisha 765022 India; 4 School of Computing and Faculty of Biological Sciences, University of Leeds, Leeds, LS2 9JT UK; 5 School of Life Sciences, College of Science, University of Lincoln, Lincoln, LN6 7TS UK

**Keywords:** Computational biophysics, Applied mathematics, Biological physics

## Abstract

The devastating trail of Covid-19 is characterized by one of the highest mortality-to-infected ratio for a pandemic. Restricted therapeutic and early-stage vaccination still renders social exclusion through lockdown as the key containment mode.To understand the dynamics, we propose PHIRVD, a mechanistic infection propagation model that Machine Learns (Bayesian Markov Chain Monte Carlo) the evolution of six infection stages, namely healthy susceptible (*H*), predisposed comorbid susceptible (*P*), infected (*I*), recovered (*R*), herd immunized (*V*) and mortality (*D*), providing a highly reliable mortality prediction profile for 18 countries at varying stages of lockdown. Training data between 10 February to 29 June 2020, PHIRVD can accurately predict mortality profile up to November 2020, including the second wave kinetics. The model also suggests mortality-to-infection ratio as a more dynamic pandemic descriptor, substituting reproduction number. PHIRVD establishes the importance of early and prolonged but strategic lockdown to contain future relapse, complementing futuristic vaccine impact.

## Introduction

Deadlier than most pandemics in the last 100 years, barring HIV and plague, Covid-19 rages on despite imposition of movement restrictions as well as clinical testing and community health measures^1,2^. As of 4 August 2020, SARS-COV-2 has infected ca 18.5 million worldwide with ca 700,000 dead. Covid-19 containment has been a major strategic issue for governments worldwide, with particular emphasis on the correct lockdown timing and span. Alarming belated infection spurt have been registered in over-populated countries like India, Brazil and Iran with early and extensive lockdowns. While the low mortality rates exhibited by low-resourced yet densely populated Asian countries have been attributed to the relative youth of the populations^3^, sparsely populated Sweden depicts an alarming dead-to-infected ratio in contrast to its European neighbours^4^.

Quarantine has been advised as the best infection control measure^5,6^. This has led to key questions as to the ideal start point and the absolute span of the ensuing lockdown. Major cases in support of lockdown are Vietnam and Cuba, that have claimed almost no death^7,8^, although such claims have been questioned^9^. In countries like Italy, the UK, the US, Sweden and Brazil, with strategic reluctance for early lockdown, comparatively softer prohibition lockdown protocols have admittedly transpired to worrisome statistics. On the other hand, European countries like Germany, the Netherlands, Belgium and France as also non-European countries like Australia, New Zealand and Korea who enforced early lockdowns initially registered remarkably low infection and mortality rates^10^, with $1.0<R_0<2.0$ during lockdown, that spiked later (www.worldometers.info). Many suffered from re-infection relapse^11,12^ with a sudden spurt in infection^13^. Regions like India, Iran and New York State, with variable quarantine measures, have all seen late infection surges. While India resorted to an early clampdown with an early withdrawal, New York State resorted to a late lockdown, but both with similar numerical implications, a feature attributed to inevitable movement of migrant workers^14^.

Analyzes of the SARS epidemic of 2003 showed that case isolation and contact tracing^1,15^, while highly effective if implemented at early stages, become ineffectual if the basic infection spread occurs before symptomatic detection^16,17^. This finding was revisited in Covid-19 transmission kinetics^18^ pointing to the importance of appropriate early (pre-symptomatic) stage strategizing. Other studies stress the importance of combining isolation^19^, social distancing with widespread testing^20^ and contact tracing^2^. Initial predictive models^14^ used data from Wuhan and Italy^20^. Both efforts suffer from a lack of robustness due to inaccurate future prediction that is reliant on sparse data, devoid of any inherent ML training protocol to emphasize on prediction rather than on data fitting.The first predictive study used a Bayesian inference structure on a simplistic SIRV model^21,22^, using infection statistics from Germany. While a move in the right direction, it suffered from two key deficiencies: lack of a time evolving death rate as an independent dynamical variable and over-reliance on infection statistics in predicting mortality rate.^20^addressed this, but it lacked the probabilistic kernel of^21^. Another issue that has often been overlooked is the best possible containment strategy in coping with the disease. Standard approaches include social distancing^23^, contact tracing^24^, social seclusion between comorbid and healthy, self- quarantine of the infected (including asymptomatic). The target in all of these is to block the epidemic spread network so that the infection chain can be broken^25^.

Vaccines have led the fight against COVID-19^26^. Multiple vaccines are now available for public use that use differential chemical pathways, e.g. mRNA replication (Pfizer^27^, Moderna^28^), viral vectors (Oxford-AstraZeneca^29^, Sputnik V^30^), antibody formation through attachment to spike proteins (Covaxin^31^, Sinofarm^32^), double-stranded DNA cloning (Janssen^33^), genetic engineering of the SARS-Cov-2 spike proteins (Novavax^34^). The vaccine arsenal is fast getting reinforced with newer additions, all targeted to mitigate the viral load as also to provide long term immunity. While expected to be a major immunity booster going forward, given the expected timeframes of vaccine rollout and perceived mutation towards newer strains of the virus (e.g. Indian variant B.1.617^35^, South African B.1.351^36^) that have at times restricted the efficacy rates of vaccines^34–36^, the major defence front will still rely on transmission mitigation through restricted movement, mask usage, sanitation codes and avoiding public gatherings, the collective impact of which could be enumerated from the PHIRVD model.

## Results

### Infection kinetics of healthy and comorbid susceptible

COVID-19 infection propagation epidemiology clearly points to the need for analyzing the vastly different infection and mortality profiles of the healthy versus the comorbid susceptible groups. Our key target is to study this interactive infection propagation and then predict future mortality and infection profiles, emphasizing mortality as the key policy indicator. The present article is to marry a robust Susceptible(S)-Infection(I)-Recovered(R)-Vaccinated(V) (SIRV) structure, estimating the reproduction number^37^, together with a Machine Learning (ML) prediction kernel, using a multi-layered error filtration structure, to generate a predictive model called PHIRVD (see “Methods” section). PHIRVD delivers three major successes at an unprecedented level of accuracy: prediction of the number of infected and dead over the next 30 days (validated using sparse data) for each of the 18 countries considered, a comparative analysis of the impact of lockdown using multiple withdrawal dates for 6 worst-hit countries with high ongoing infection rates, and a detailed temporal profile of future reproduction numbers that can be (and have been) verified against real data. PHIRVD also establishes mortality-to-infection ratio as the key dynamic pandemic descriptor instead of reproduction number.

### Mathematical model—PHIRVD

**Figure 1:**
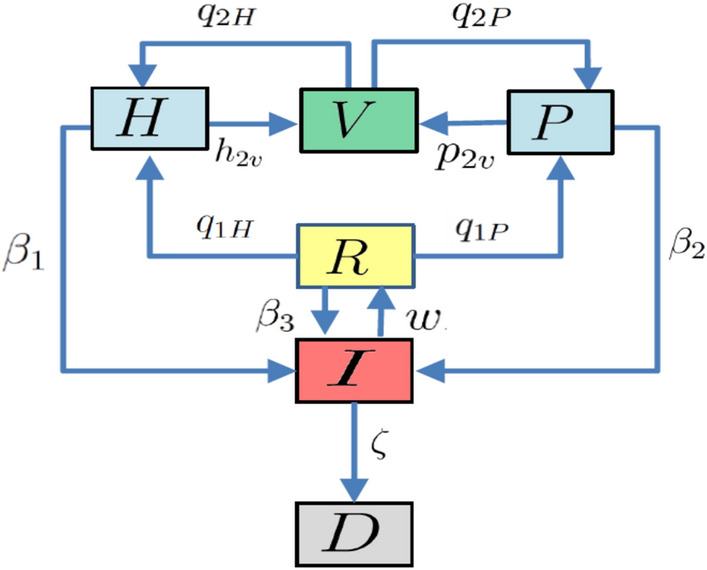
Schematic diagram outlining the infection kinetic profile of our model PHIRVD: healthy susceptible (*H*); predisposed comorbid susceptible (*P*); infected (*I*); recovered (*R*); herd immunized (*V*) and dead (*D*).

Our compartmentalised Covid-19 pandemic kinetics uses a 6-dimensional dynamical system as in Eq. (1), combining SIR and SEIR kernels^38,39^, schematically outlined in Fig. 1:

1 $$\begin{aligned} \frac{dH}{dt}= & {} -\beta _1 H I {+\ q_{1H}R + q_{2H}V } - {h_{2v} H} - \gamma H, \nonumber \\ \frac{dP}{dt}= & {} -\beta _2 P I -(\gamma +\delta ) P {+\ q_{1P}R + q_{2P}V }-p_{2v} P, \nonumber \\ \frac{dI}{dt}= & {} (\beta _1 H + \beta _2 P + \beta _3 R) I -(\gamma +\zeta ) I - w I, \nonumber \\ \frac{dR}{dt}= & {} w I - \beta _3 R I - \gamma R - q_{1H} R - q_{1P}R, \nonumber \\ \frac{dV}{dt}= & {} {-(q_{2H}+q_{2P})V } -\gamma V + h_{2v} H + p_{2v} P, \nonumber \\ \frac{dD}{dt}= & {} \gamma (H+R+V) + (\gamma +\delta )P + (\gamma +\zeta ) I. \end{aligned}$$

The parameters in this model, that we call PHIRVD, characterize the infection rate of healthy agents ($\beta _1$), infection rate of agents with pre-existing health conditions ($\beta _2$), relapse rate ($\beta _3$), conversion rates of recovered to healthy susceptible ($q_{1H}$) and previously “immuned” to healthy susceptible ($q_{2H}$), conversion rates of recovered to pre-existing susceptible ($q_{1P}$) and previously “immuned” to pre-existing susceptible ($q_{2P}$), death rate due to non-Covid interference ($\gamma$), additional death rate due to agents with pre-existing conditions ($\delta$) and that due to infected ($\zeta$), recovery rate (*w*), rate at which healthy ($h_{2v}$) and pre-existing susceptible ($p_{2v}$) groups are quarantined. Our focus being Covid-19 infection and mortality statistics, we neglect death rate ($\gamma =0$) and additional death rate ($\delta =0$) due to all non-Covid causes. Since death rate of healthy infected is a lot lower than that of the comorbid and elderly death rate (https://www.cdc.gov/coronavirus/2019-ncov/need-extra-precautions/older-adults.html)^40,41^; hence we have added a practical constraint in our model to account for this effect that expresses in the form of $\beta _1<\beta _2$. Hence, the infection rate of *H*-group is considered to be a small fraction ($\lambda$) of the *P*-group, i.e. $\beta _1=\beta _2 \lambda$. The death variable *D* thus acts like a “sink” of the dynamical system ensuring a population conservation inbuilt within the model ($H+P+I+R+V+D$ = constant). The PHIRVD model can be easily extended to incorporate the impact of upcoming and available vaccines. The impact points would be at the transitory phases between prolonged lockdown, characterized by low susceptible-infected coupling, to a lockdown withdrawal, typically leading to a surge in the infection/mortality traffic, a case of human reaction to maximize social expression.

In training our model, we find it useful to define an extra variable $I_c(t)$, which represents the cumulative number of those infected upto a given date. In other words, it includes not only those who are currently infected, but also those who have since recovered or died, *i. e.* $\frac{dI_c}{dt}=(\beta _1 H \beta _2 P + \beta _3 R) I.$ Since we have considered relapse in our model, it is to be noted that $I_c(t) \ne I(t) + R(t) + D(t)$.

### Data repositories

Identifying the infection kinetics of Covid-19 as an interactive evolution process involving six time evolving population density variables: healthy susceptible (*H*), susceptible with pre-existing conditions or comorbidity (*P*), infected (*I*), recovered (*R*), naturally immuned (i.e. a clone for vaccinated *V*) and dead (*D*), the PHIRVD model uses statistics from the Johns Hopkins Covid-19 database^42^ to accurately predict mortality and infection statistics of 18 Asian, European and American countries. Data threshold was set beyond the first 19 days of low (or no) infection, followed by data training between 10 February 2020 to 29 June 2020. Results were later cross-verified from other databases e.g. US: https://usafacts.org; EU: https://data.europa.eu/; UK: https://coronavirus.data.gov.uk/; India: https://www.covid19india.org/. The Bayesian Markov Chain Monte Carlo (MCMC)^43^ infrastructure in PHIRVD trains the repository data to probabilistically predict the 17 parameters of the infection kinetic model (see “Methods” section). Unlike previous predictive Machine Learning models^14,19–22^, this structure allows more dynamic adaptive control of the infection kinetic estimation resulting in a highly accurate predictive module.

### Mortality and infection: prediction against reality

The 18 countries or regions under study were divided into 4 infection classes, the first three based on decreasing mortality-to-infection ratio for countries past their infection peak: UK, Netherlands, Sweden, New York State (**Class A**); Germany, Korea, Australia, Russia, Vietnam (**Class B**); and Italy, Spain, Hubei (**Class C**). Class **Class D** comprises India, Poland, Iran, France, Portugal and Brazil, with ongoing infection regimes. We deliberately chose New York State instead of the entire United States due to its high population density and tourist/ worker traffic that is quite different from the national average.

**Figure 2:**
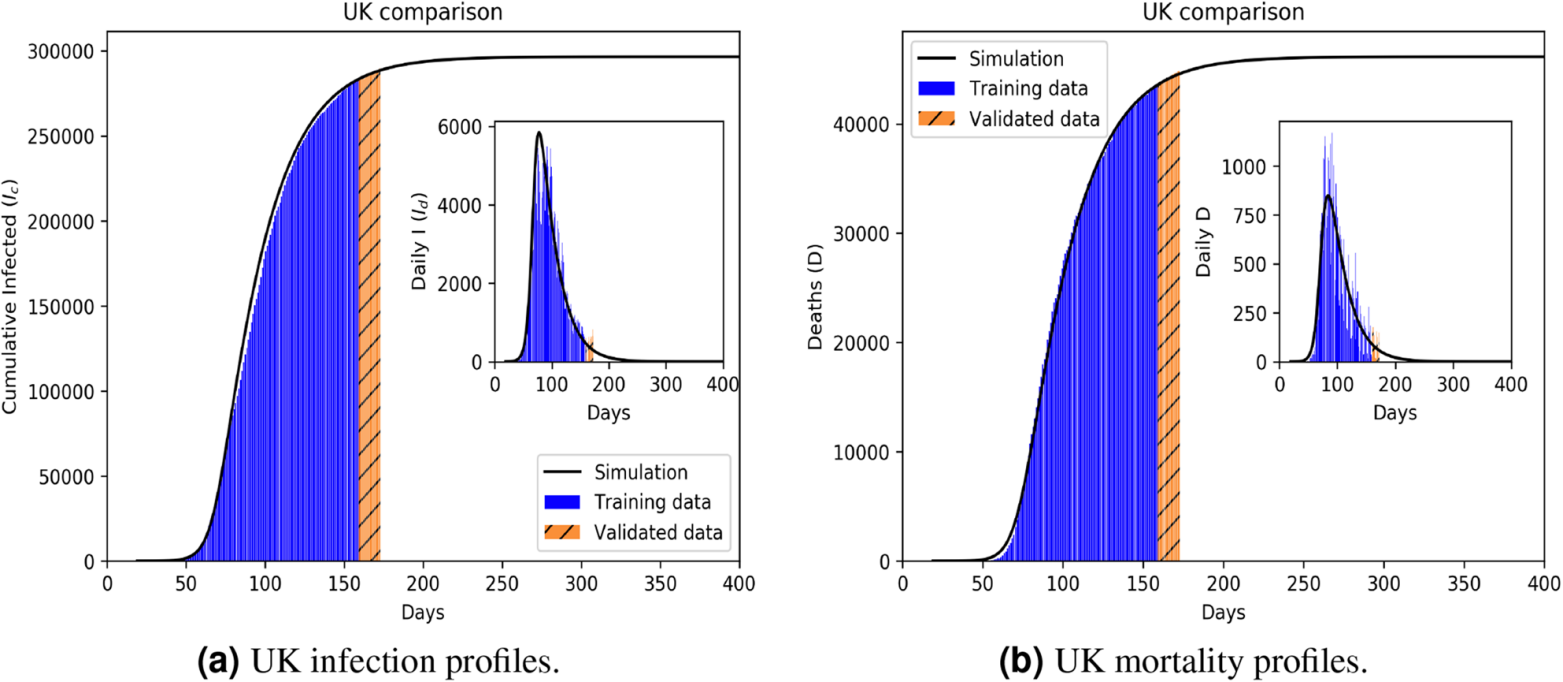
Infection (**a**) and mortality (**b**) epidemiology for the UK (Class A). Outsets represent cumulative statistics while the insets are for daily updates in the number of infected and death respectively. Here “0” marks 22 January 2020; data training between 10 February to 29 June 2020.

**Figure 3:**
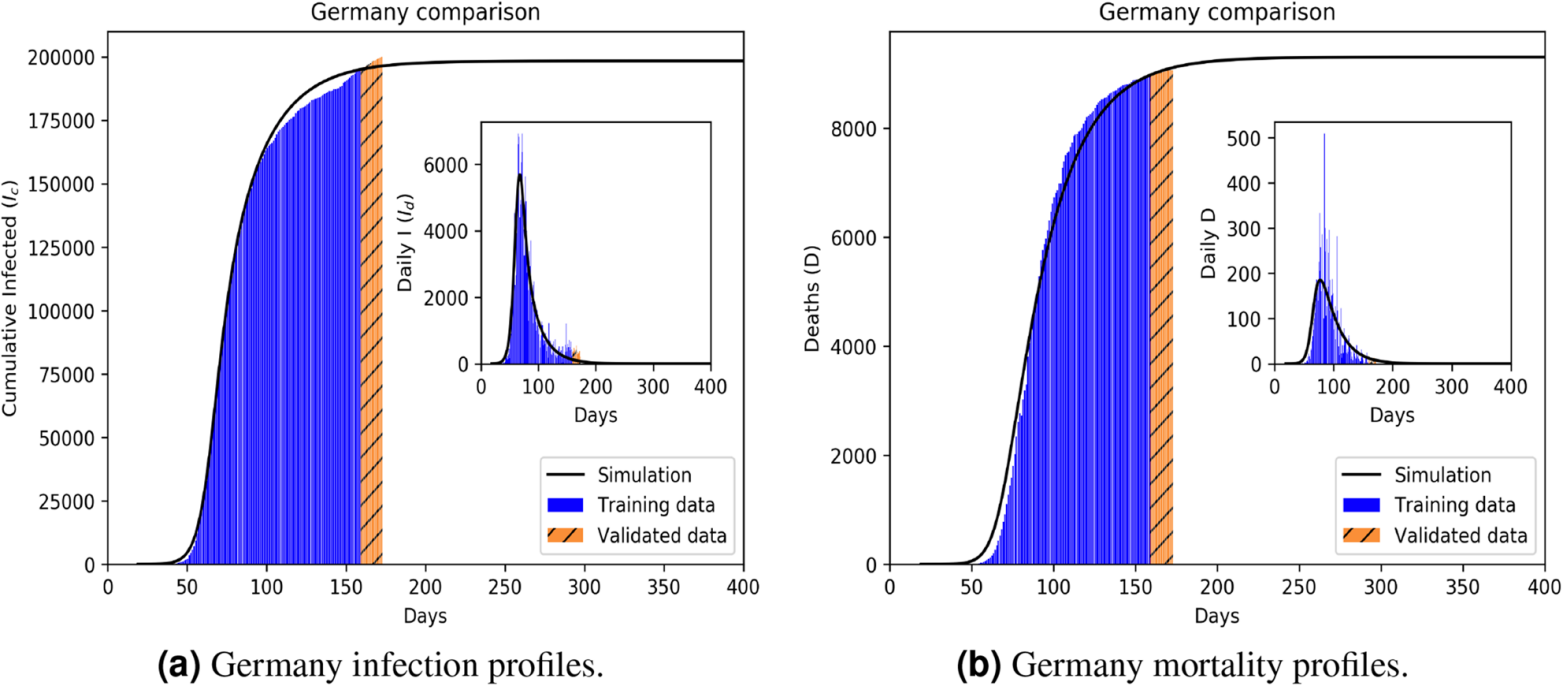
Infection (**a**) and mortality (**b**) epidemiology for Germany (Class B). Outsets represent cumulative statistics while the insets are for daily updates in the number of infected and death respectively. Here “0” marks 22 January 2020; data training between 10 February to 29 June 2020.

**Figure 4:**
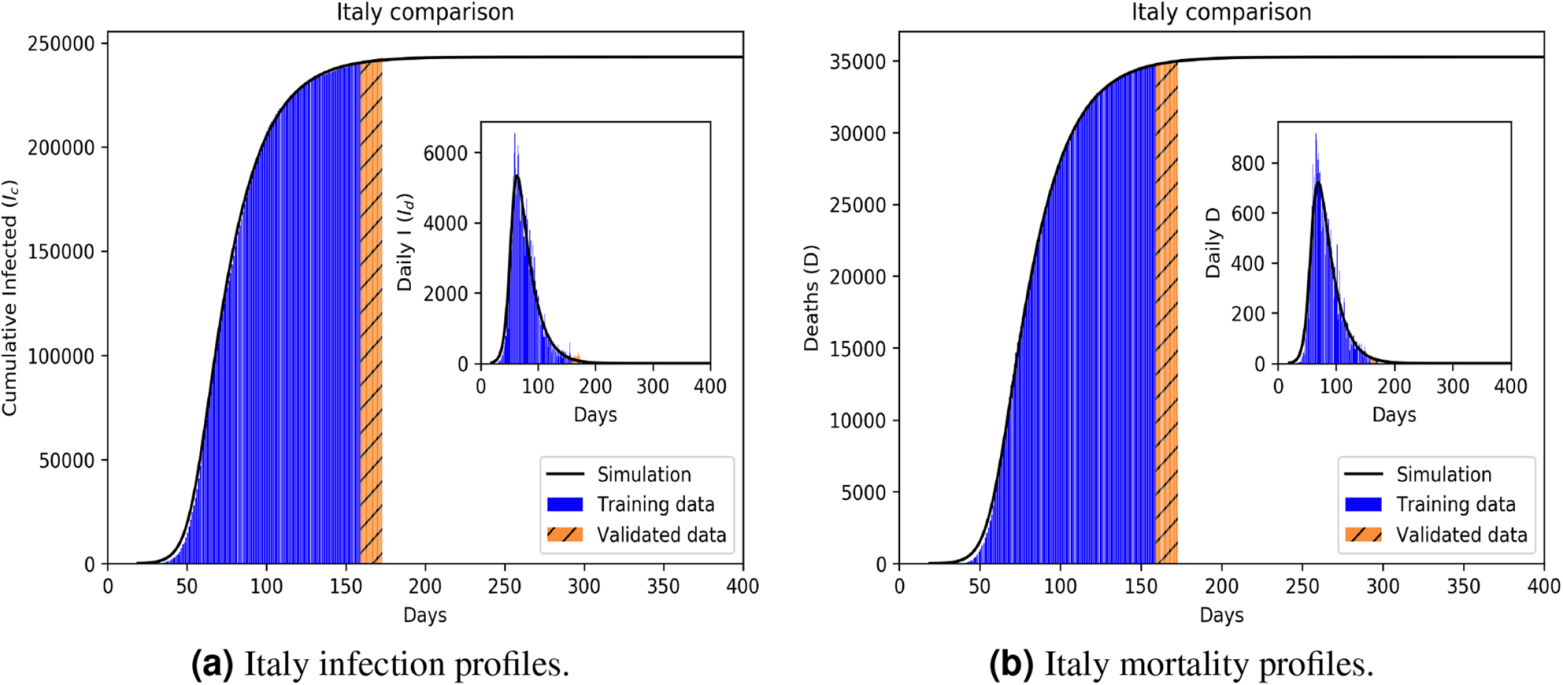
Infection (**a**) and mortality (**b**) epidemiology for Italy (Class C). Outsets represent cumulative statistics while the insets are for daily updates in the number of infected and death respectively. Here “0” marks 22 January 2020; data training between 10 February to 29 June 2020.

**Figure 5:**
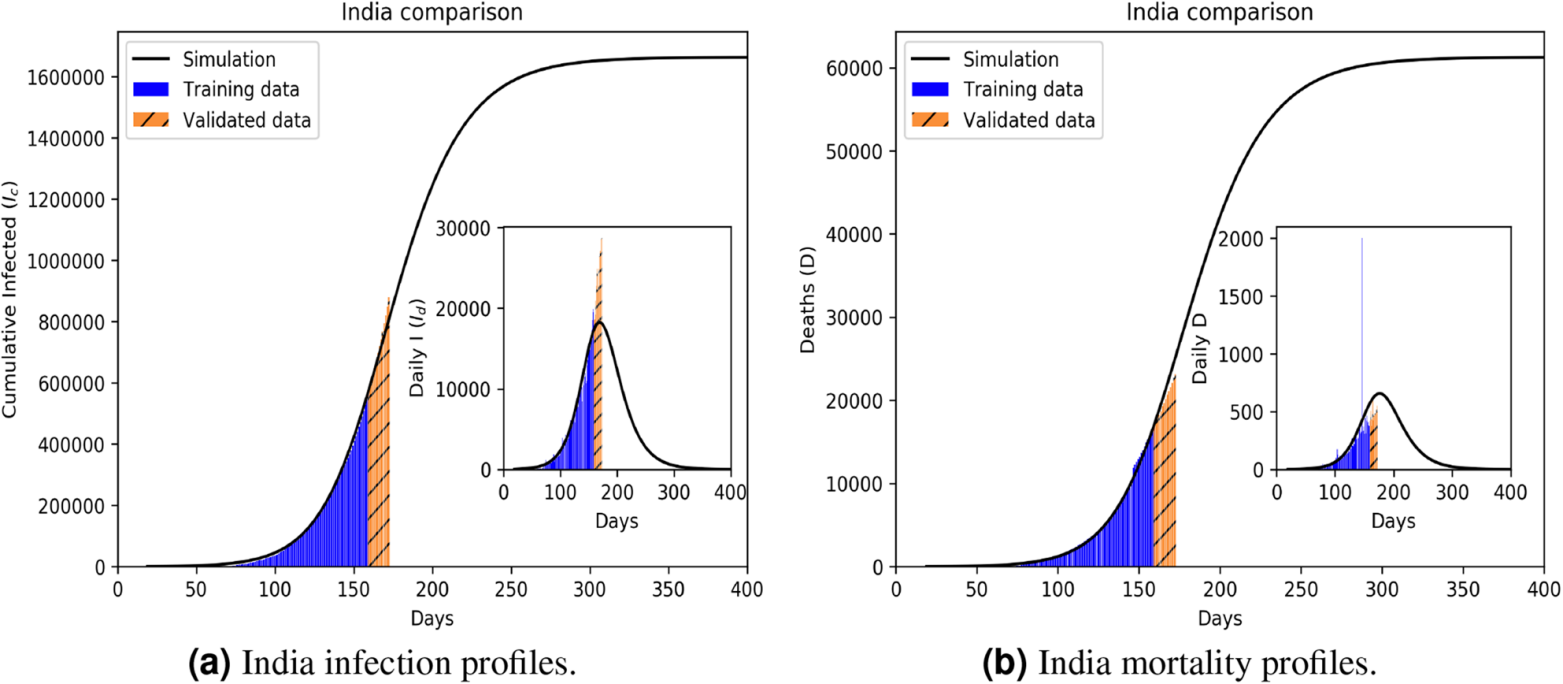
Infection (**a**) and mortality (**b**) epidemiology for India (Class D). Outsets represent cumulative statistics while the insets are for daily updates in the number of infected and death respectively. Here “0” marks 22 January 2020; data training between 10 February to 29 June 2020.

With the number of reported cases being highly dependent on the number of daily testings, not necessarily in agreement with the actual disease propagation dynamics, we observe some deviations between the simulated *I*(*t*) and the actual number of reported cases. On the other hand, *D*(*t*) is less affected by the testing rate. Since we are using mortality statistics with the same weightage as the infected data, we prioritize mortality prediction. We note that daily training of any epidemiological model will invariably achieve better data match, as many studies have shown. However, our ML embedded propagation kinetic model thrives on long term predictions, as much as possible.

Comparative statistics for our Class A representative, the UK, is shown in Fig. 2. The blue region marks the training zone that fixes the parameters. Based on the highest mortality to infection ratio in each group, the representative countries for the other 3 classes are Germany (Class B), Italy (Class C), India (Class D). Figures 3, 4 and 5 represent infection statistics for Class B (Germany), C (Italy) and D (India) respectively (other plots in Appendix II). Chi-square tested (see “Methods” section for Chi-squared statistic used) accuracy chart in Table 1 clearly points to the veracity of the accuracy claim made. On the other hand, Vietnam presents an interesting case. With a reported zero mortality rate notwithstanding high population density, it has been repeatedly cited as an example of early quarantine success. The model tracks even such an exceptional case to a moderate level of accuracy (in Appendix II). The outsets and insets respectively outline the cumulative versus the daily infection traffic. Details for other countries, for 4 infection classes, are provided in Appendix II.

Table 2 presents a comparative chart of the PHIRVD model predictions versus real data, separately for the numbers of infected and dead, for countries representing the 4 classes with data trained between 10 February to 29 June: Class A (UK), Class B (Germany), Class C (Italy) and Class D (India). Futuristic prediction is shown until 12 July. For other countries in each individual class, with data training between 10 February to 10 May, 30 days’ prediction until 9 June establishes the predictive strength of this model (see Tables S2–S5, Appendix III), error validated as shown in Table S1 (see Appendix I).

**Table 1:** *p* Values for daily new infected and dead for Class A–D representative countries between 11 Feb to 16 June 2020.

Country	Daily new infected		Daily new death	
	$\epsilon$	*p* value	$\epsilon$	*p* value
UK	0.26	0.23	0.36	0.14
Germany	0.42	0.18	0.45	0.25
Italy	0.32	0.22	0.3	0.28
India	0.52	0.25	0.52	0.38

**Table 2:** Validation of daily new infected and death: UK (Class A), Germany (Class B), Italy (Class C), India (Class D).

Days	Country															
	UK				Germany				Italy				India			
	Infected		Death		Infected		Death		Infected		Death		Infected		Death	
	Data	Simulation	Data	Simulation	Data	Simulation	Data	Simulation	Data	Simulation	Data	Simulation	Data	Simulation	Data	Simulation
30/06/20	403	472	155	91	376	126	14	12	142	121	23	23	18641	17298	507	570
01/07/20	60	455	176	88	475	121	5	11	182	116	21	22	19160	17471	434	579
02/07/20	4	439	89	84	477	117	11	11	201	110	30	21	20903	17628	379	588
03/07/20	502	424	136	81	410	112	4	11	223	106	15	20	22771	17767	442	596
04/07/20	624	408	67	79	418	108	10	10	235	101	21	19	24850	17889	613	604
05/07/20	516	394	22	76	325	104	3	10	192	96	7	18	24248	17992	425	612
06/07/20	352	380	16	73	541	100	0	9	208	92	8	17	22251	18078	466	618
07/07/20	581	366	155	70	279	96	10	9	137	88	30	17	22753	18145	483	625
08/07/20	630	353	126	68	356	92	14	9	193	84	15	16	24879	18194	487	631
09/07/20	642	341	85	66	302	89	11	8	214	81	12	15	26506	18224	475	636
10/07/20	512	329	48	63	331	85	6	8	276	77	12	14	27114	18236	519	640
11/07/20	820	317	148	61	377	82	7	8	188	74	7	14	28606	18230	550	645
12/07/20	650	306	21	59	210	79	1	7	234	70	9	13	28732	18206	501	648

Table 3 compares second wave mortality prediction obtained from PHIRVD against real data, based on data training until 29 June 2020. The result can be substantially improved if data is trained within a month of the resurgent wave, as in November 2020. But the reliability of prediction stretching up to 150 days beyond last data training is unprecedented to our knowledge and affirms the robustness of the model.

**Table 3:** UK second-wave validation for mortality data.

UK validation-death profile		
Date	Real data	PHIRVD prediction
10/11/20	595	457
11/11/20	563	448
12/11/20	376	439
13/11/20	462	430
14/11/20	168	420
15/11/20	213	411
16/11/20	598	401
17/11/20	529	392

**Figure 6:**
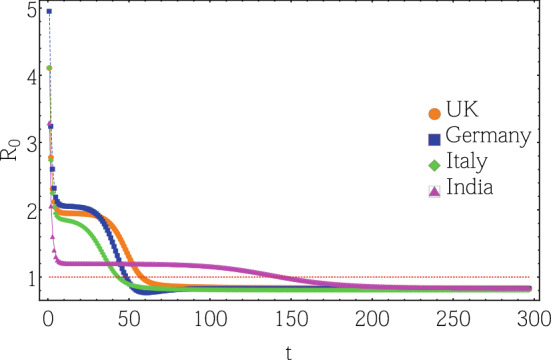
Daily temporal evolution of the basic reproduction rate for countries from Class A (UK), Class B (Germany), Class C (Italy) and Class D (India). The dotted line sets the pandemic threshold; count “0” starts at 14 February 2020, excluding data for the first 19 days (statistics recorded 22 January 2020 onwards) due to low infection, and additional 4 days of generation time. MCMC training between 10 February 2020 to 29 June 2020.

The expected number of secondary cases produced from each infected individual is traditionally defined as the basic reproduction number. The detailed calculation of $R_{\text {e}}$ is provided in the “Methods” section. Figure 6 depicts the time evolution of basic reproduction number that indirectly reflects the emerging infection (and fatality) rate for the 4 representative countries from infection classes A-D, represented by the basic reproduction number $R_0$^44–46^ (see “Methods” section). $R_0$ kinetics of all other countries are provided in Appendix I. Class A countries consistently show the sharpest drop in $R_0$ and the flattest stability period, followed by progressive R0 decay and waiting time, often the ‘gestation time’, reflected by the plateau regions of the respective plots for classes B, C and D respectively. The point of note here is that while Germany and Italy show higher levels of infection than the UK, the gestation period for the UK is a lot larger than both. India shows a similar trend although the absolute numbers for India are a lot lower than the other three, indicating a complicated relationship between Full Width at Half Maximum (FWHM) and gestation period.

## Discussion

Combining conventional infection kinetic modeling with a predictive Bayesian MCMC, PHIRVD quantifies the impact of lockdown as a containment tool. It estimates mortality statistics with high significance for 18 countries, accurate upto the next 30 days, beyond the last date of data training. Ideal lockdown imposition and withdrawal times have been predicted and validated, including for ongoing regimen e.g. India. PHIRVD also predicts secondary relapse timings and establishes mortality-to-infection ratio as the key pandemic predictive descriptor instead of reproduction number. PHIRVD is also capable of analyzing the impact of migration, an ongoing project. Our findings clearly suggest that phased lockdown is a potent containment tool but needs to be strategically imposed, where the correct implementation and withdrawal times are paramount. Secondary infection and mortality prediction will be key to future strategic quarantine imposition and analyzing impact of future therapeutics.

PHIRVD leads to three key outcomes. First, we present highly accurate probabilistic predictions for the numbers of infected and dead for each country for a total of 18 countries, typically 3 weeks beyond the last date of (Machine Learned) data training. Our PHIRVD model depicts a high degree of reliability between model prediction against real data validation across the range of countries considered.

**Figure 7:**
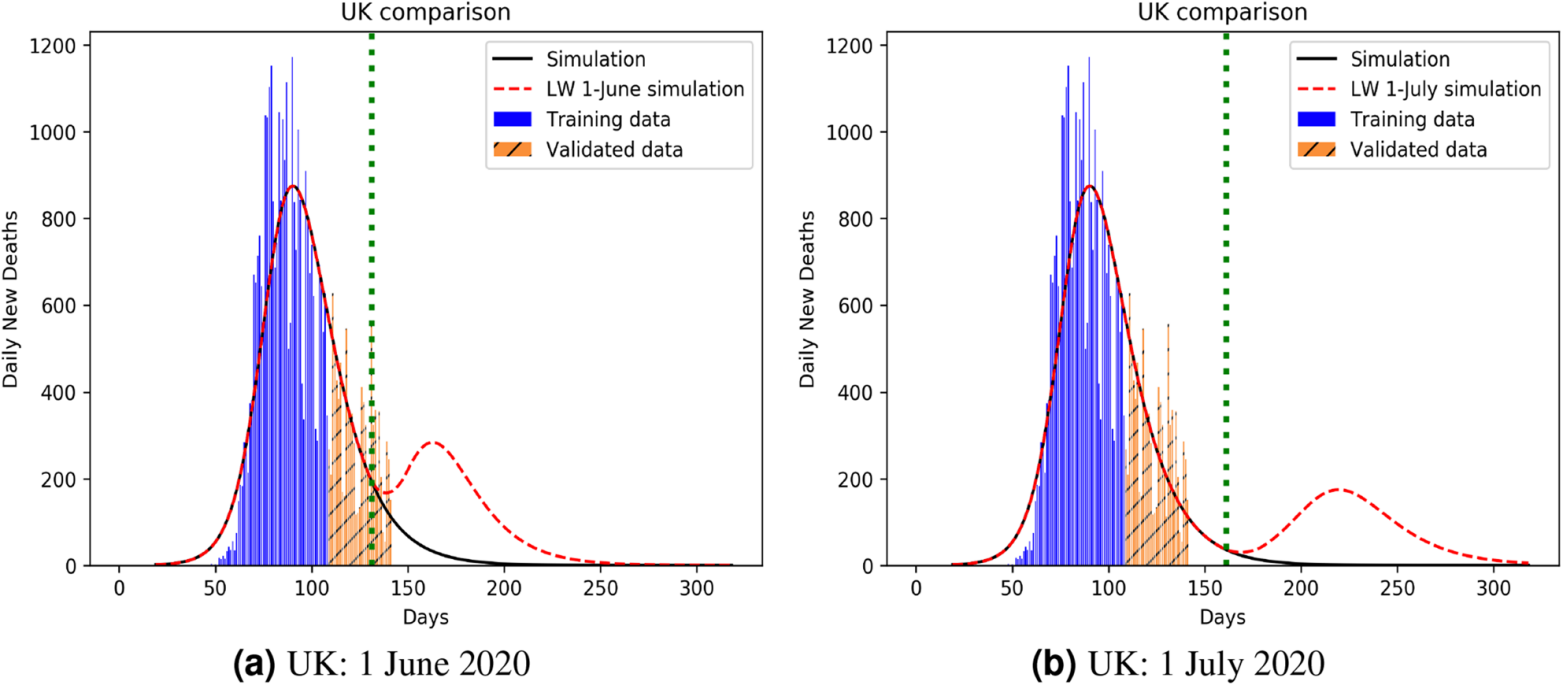
Infection kinetics compared for the UK for different lockdown withdrawal dates. Analysis is based on daily mortality statistics. The vertical dotted line represents lockdown withdrawal date $t_0$ in the function *L*(*t*) (see “Lockdown Dynamics” in “Methods” section). For the left and right panels $t_0$ is set to be 1 June 2020 and 1 July 2020, respectively. $k=20$ and $\alpha =2.4$ are used in the function *L*(*t*) for simulating red dashed curves in both the plots. Here “0” marks 22 January 2020.

**Figure 8:**
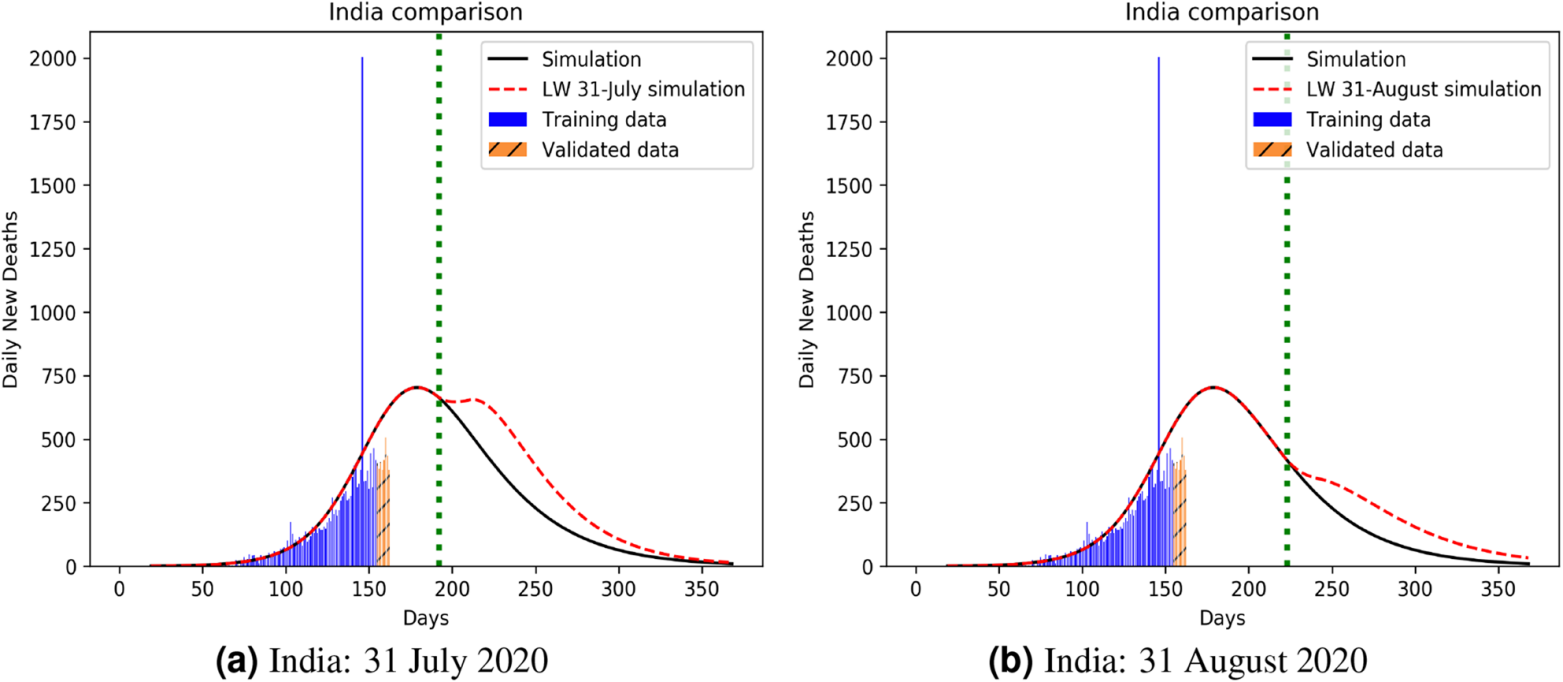
Infection kinetics compared for India for different lockdown withdrawal dates. Analysis is based on daily mortality statistics. The vertical dotted line represents lockdown withdrawal date $t_0$ in the function *L*(*t*) (see “Lockdown Dynamics” in “Methods” section). For the left and right panels $t_0$ is set to be 31 July 2020 and 31 August 2020, respectively. $k=20$ and $\alpha =1.9$ are used in the function *L*(*t*) for simulating red dashed curves in both the plots. Here “0” marks 22 January 2020.

Our model can also be used to identify a better strategy for lockdown imposition, to minimize the fatality. The full simulations plots (in Appendix II) clearly outlines how an increasing infection profile initially matches with decreasing numbers of pre-existing susceptible and increasing statistics for the recovered, that then slows down as the infection peak arrives, eventually to tail off in to a no-infection landscape. While the qualitative trends are similar for all classes (A, B, C, D) of countries, the impact of lockdown on the first peak, and then a second (relapse) peak, hint at the internal health versus econometrics of the countries concerned. To prove this point, we compare infection (and mortality) propagation kinetics of 2 chosen countries for two different dates, one on the recess (UK: Fig. 7), the other with uprising infection level (India: Fig. 8). As opposed to the recent furore about school children being exposed to the Covid-19 menace as a result of early lockdown withdrawal, our result clearly shows that there is practically no difference in mortality between a withdrawal on June 1, 2020 as against a later withdrawal e.g. July 1, 2020 (although a withdrawal on May 1 would have been disastrous). The 1 June (almost equally safe) withdrawal would, of course, be favoured on economic and social grounds.

**Figure 9:**
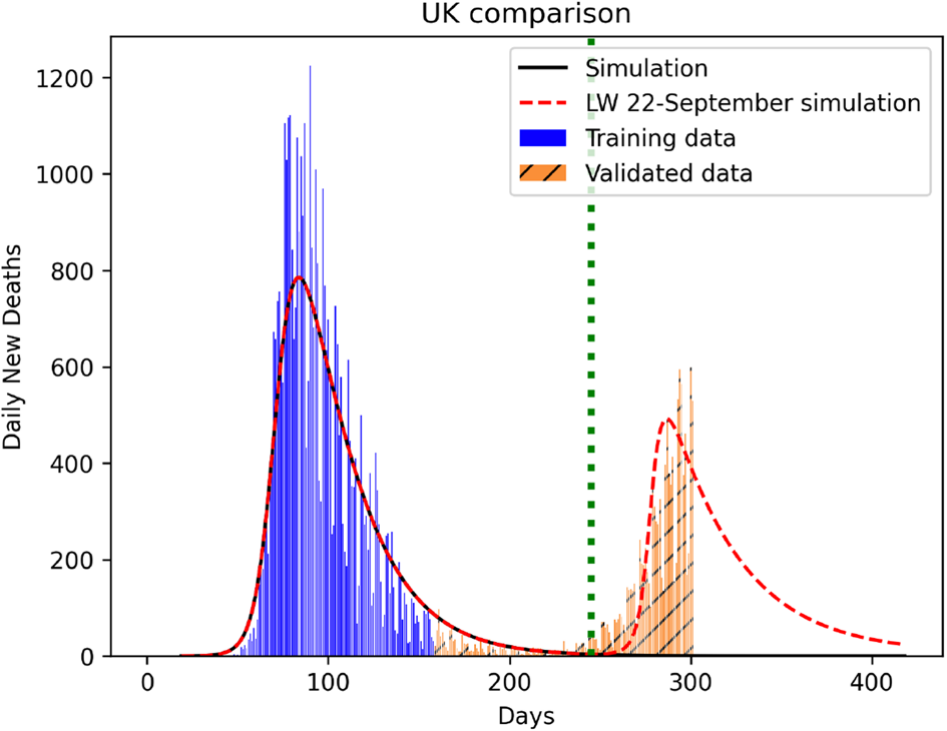
Prediction of infection resurgence (second wave) in the UK from PHIRVD. Count “0” starts at 22 January 2020. MCMC training performed between 10 February 2020 to 29 June 2020, excluding data for the first 19 days (statistics recorded 22 January 2020 onwards) due to low infection. The second wave is simulated by setting $t_0=$ 22 September 2020 (marked by the vertical dotted line), $k=450$, and $\alpha =35$, in the function *L*(*t*) (see “Lockdown Dynamics” in “Methods” section).

The third key outcome of our analysis is the establishment of mortality:infection ratio as the key descriptor of pandemic over and above reproduction number, that has conventionally been used for the purpose. The proof of this is in the accurate prediction of the secondary infection relapse time that the reproductive number fails to predict. As can be seen from Fig. 7a,b, this relapse time period could be deferred with a late lockdown withdrawal on July 1 (as compared to June 1) although the peak mortality rates are not hugely different (ca 200 at 1 July compared to ca 400 at 1 June). Using 1 July 2020 as the UK lockdown withdrawal date, there is a clear signature of secondary relapse in the first week of September (identified as the second peak in Fig. 7). The Indian situation is clearly more challenging, though, as shown in Fig. 8. While perhaps economically unsustainable, India could benefit with a lockdown even beyond 31 July, 2020. For other nations like Iran, Portugal, France and Poland, our predictions of non-trivial secondary relapses (all in late June) match almost perfectly with data, both infected and dead. As the second wave data is now available for the UK, we simulated it using our PHIRVD model. Results shown in Fig. 9 demonstrate excellent agreement with real statistics (data trained only up to 29 June 2020), that reaffirms the strength of the model.

A real point of contention amongst politicians, health professionals and medical scientists has, for long, been the correct lockdown implementation and withdrawal times. In statistical parlance, this effectively amounts to an estimation of the FWHM as has been estimated for Wuhan at 2.6 weeks from initial infection^47^. To analyze these counterclaims, we incorporate the effects of withdrawal of lockdown as a country specific, dynamically evolving quantity.

The availability of the awaited vaccines^26^, and of late, the therapeutic range^48,49^, have provided major immunity tools in the Covid firefight. The impacts of these vaccines are most likely to be futuristic antibody switch though, as is clearly evidenced by the huge second/third phase outbreaks in countries like India, Bangladesh and Russia that survived the initial onslaught well. With growing mortality profile, sometimes attributed to newer viral strains, the impact of quarantine measures, namely what and how to choose and when to implement or withdraw, has now assumed crucial importance, for which our model can serve as a future benchmark.

## Methods

### Motivation of the PHIRVD model

PHIRVD uniquely combines a dynamically evolving infection propagation model that tracks the phenomenology of infection kinetics with a probabilistic predictive algorithm, the latter chosen as a Bayesian Markov Chain Monte Carlo (MCMC) kernel. The Bayesian MCMC is used to train past data to predict time independent generic parameters that can predict the future statistics. The choice is guided by the strength of Bayesian MCMC in a range of dynamical modeling studies in complementary fields^50,51^.

### Reproduction number $R_{\text {e}}$ at fixed point

For $\gamma =0, \delta =0$, from Eq. (1) the disease free equilibrium (DFE) or fixed point is given by $P^* =H^*\frac{h_{2v} q_{2P}}{p_{2v} q_{2H}}$, $I^*=0, R^*=0$, $V^*=H^* \frac{h_{2v}}{ q_{2H}}$. To evaluate the reproduction number $R_{\text {e}}$, we have to break the equation of $\frac{dI}{dt}$ into two parts ${\mathcal {F}}, {\mathcal {V}}$, i.e.,

2 $$\begin{aligned} \frac{dI}{dt}={\mathcal {F}}-{\mathcal {V}} \end{aligned}$$

where ${\mathcal {F}}=(\beta _1 H + \beta _2 P + \beta _3 R) I$ and ${\mathcal {V}}=(\zeta +w) I$. Now, $F=\frac{\partial {\mathcal {F}}}{\partial I}|_{DFE}$ and $\Sigma =\frac{\partial {\mathcal {V}}}{\partial I}|_{DFE}$. Then $R_{\text {e}}=\frac{F}{\Sigma }=\frac{ H^* \left( \frac{\beta _{2} h_{2v} q_{2P}}{p_{2v} q_{2H}}+\beta _{1}\right) }{\zeta +\omega }$.

### Lockdown dynamics

During the time period, over which we trained our model, most of the countries (except Sweden), of our interest, were under lockdown. Therefore, we studied the effects of withdrawal/relaxation of lockdown for some countries by introducing a time varying parameter *L*(*t*) in the model in Eq. (1) substituting $\beta _{1,2,3}$ with $\beta _{1,2,3}\,L(t)$ respectively, where $L(t) = 1 \,\,\text {for} \,\,t \le t_0, \,\,\text {and}\,\,\alpha \,\,\text {for}\,\,t \ge t_0+k$. For $t_0<t<t_0+k$, $L(t) = \frac{1}{k} [ \alpha (t-t_0)+(t_0+k-t) ]$. Here $t_0$ marks the lockdown withdrawal time point, *k* is the approximate time duration during which the susceptible and infected population mixes well (e.g. within one week or one month etc.), where $\alpha$ is the parameter quantifying the intensity of mixing between susceptible and infected population. A larger $\alpha$ value implies a higher mixing rate among susceptible and infected individuals. The function *L*(*t*) is such that before lockdown withdrawal, it does not alter the contact probability while after withdrawal, it linearly increases from the value 1 to $\alpha$ over a time interval of *k* days, ensuring that the contact probability between susceptible and infected increases from a low to a high value within this time period.

### Parameter estimation

The Bayesian MCMC data training leading to supervised learning is itself conducted in two steps using a double-filtration process. First, infection data alone are used to arrive at a preliminary set of values, characterizing each country. The said values are then filtered through combined infected and mortality statistics for a second training to sequentially converge to a preset upper limit. The training schedule is repeated multiply to ensure accurate predictions of the training dataset. Estimation of the equilibrium reproduction number is strategically used to reduce the effective parameter space from 13 to 8 parameters, perfectly conforming with the Bayesian MCMC prediction which shows that value fluctuations with other parameters do not contribute much to the infection kinetics. The model clearly separates the *H* and *P* infection classes to reflect their differential levels of infection and mortality. Another constituent is the death rate kinetics embedded in the central structure. The infection propagation model outlined in Eq. (1) is a multi-parameter model whose parameters are evaluated using predictive data modeling within the Bayesian MCMC construct. Similar structures have been selectively used in^21,22^ albeit for single-country specific models without any explicit mortality dynamics. Over-reliance on infection statistics has often led to incorrect estimation for mortality statistics, whose accurate prediction is our first key target, an aim that is remarkably well served by our ML-embedded compartmentalised model. We present both the cumulative and daily (inset plots) statistics of infected population over 400 days, data trained between 10 February 2020 to 29 June 2020 (140 days) and then predicted up to the next 8 weeks (shown up to 12 July 2020 in Table 1).

### The Bayesian Markov chain Monte Carlo (MCMC) algorithm

To understand how the algorithm uses the data to determine the parameters, it is useful to recall some elements of Bayesian statistics^50,51^. Let $\varvec{D}=(D_1, D_2, \ldots , D_n)$ represent the full data vector that is being used to train the algorithm. For our case, the subscripts run over both the time intervals (daily) as well as the data types, such as $I_c(t_i)$ and $D(t_i)$. Similarly, let $\varvec{\Theta }=(\theta _1, \theta _2, \ldots , \theta _\alpha )$ represent the vector of parameters. A key ingredient is the prior probabibility distribution (*Bayesian priors*) for each $\theta _i$. While the absence of any knowledge of the system would call for a prior that is flat in the physically allowed region, the incorporation of such knowledge (which, in the present context, could be divined from the analysis of, say even part of the data for a single country in a given class) quickly gives the prior a somewhat peaked structure. In other words, one could as well start with a normal-distributed prior, *viz.*, $\varvec{\Theta } \sim N(\varvec{\Theta _0,\sigma })$, where the vector $\varvec{\Theta _0}$ represents the mean of the parameters and $\varvec{\sigma }=(\sigma _1, \sigma _2, \ldots , \sigma _\alpha )$ the standard deviation. As it turns out, the dependence of the final result on the prior is quite insignificant.Given a $\varvec{\Theta }$, it is straightforward to calculate the conditional probability ${\mathscr {P}}(\varvec{D|\Theta })$ of obtaining a realization $\varvec{D}$ for the data. Using Bayes’ theorem, the posterior probability for $\varvec{\Theta }$ given the data is expressed as

3 $$\begin{aligned} {\mathscr {P}}(\varvec{\Theta }|D)=\frac{{\mathscr {P}}(\varvec{D|\Theta }){\mathscr {P}}(\varvec{\Theta })}{{\mathscr {P}}(\varvec{D})}, \end{aligned}$$

where ${\mathscr {P}}(\varvec{D)=\int _\Omega {\mathscr {P}}(\varvec{D}|\varvec{\Theta }}) {\mathscr {P}}(\varvec{\Theta })d\varvec{\Theta }$, with $\Omega$ denoting the whole parameter space. This, immediately leads us to the likelihood ratio of two parameter vectors $\varvec{\Theta _1}$ and $\varvec{\Theta _2}$, namely

4 $$\begin{aligned} \frac{{\mathscr {P}}(\varvec{\Theta _2|D})}{{\mathscr {P}}(\varvec{\Theta _1|D})} =\frac{{\mathscr {P}}(\varvec{D|\Theta _2}){\mathscr {P}}(\varvec{\Theta _2})}{{\mathscr {P}}(\varvec{D|\Theta _1}){\mathscr {P}}(\varvec{\Theta _1})} \ . \end{aligned}$$

We now resort to a 3-step algorithm:

1. Choose parameters (including initial conditions) through a random walk in the parameter space. The nature of the random walk is determined by the prior probability distributions for the parameters, including initial conditions.
2. Calculate the likelihood ratio function for the parameters, given the data.
3. Decide whether to accept the suggested parameter set or not.

**Step 1:**

Let $\varvec{S_i}=(S_{i1}, S_{i2}, \ldots , S_{in})$ be the simulated vector at the *i*th step for parameter values $\varvec{\Theta _i}=(\theta _{i1}, \theta _{i2}, \ldots , \theta _{i\alpha })$. Compared to the total population, the data $I_c(t), D(t)$ *etc.* are quasi-continuous and can be assumed to be drawn from a Normal distribution with respective standard deviations $\varvec{\Gamma }=(\gamma _1, \gamma _2, \ldots , \gamma _n)$ and means $\varvec{S_i}=(S_{i1}, S_{i2}, \ldots , S_{in})$. Therefore, the posterior probability (or likelihood, in case of continuous probability density) of the parameter vector $\varvec{\Theta _i}$ is,

5 $$\begin{aligned} \displaystyle {\mathscr {P}}(\varvec{\Theta _i|D})=\frac{{\mathscr {P}}(\varvec{D|\Theta _i}) {\mathscr {P}}(\varvec{\Theta _i})}{{\mathscr {P}}(\varvec{D})} = (2\pi )^{-(n+\alpha )/2} \left[ \prod _{j=1}^n\gamma _j\prod _{\beta =1}^\alpha \sigma _\beta {\mathscr {P}}(\varvec{D})\right] ^{-1} \, \exp \left( \frac{-1}{2}\sum _{j=1}^n\left( \frac{S_{ij}-D_j}{\gamma _j}\right) ^2\right).\end{aligned}$$

Next, we execute a random walk in $\varvec{\Theta }$-space with distribution $N(\varvec{\Theta _i,\sigma })$ to find $\varvec{\Theta _{i+1}}$, and calculate again the posterior likelihood function, with the simulated data vector $\varvec{S_{i+1}}$, corresponding to the parameter vector $\varvec{\Theta _{i+1}}$ as

6 $$\begin{aligned} \displaystyle {\mathscr {P}}(\varvec{\Theta _{i+1}|D})= & {} \displaystyle \frac{{\mathscr {P}}(\varvec{D|\Theta _{i+1}}) {\mathscr {P}}(\varvec{\Theta _{i+1}})}{{\mathscr {P}}(\varvec{D})} \nonumber \\= & {} \displaystyle (2\pi )^{-(n+\alpha )/2} \left[ \prod _{j=1}^n\gamma _j\prod _{\beta =1}^\alpha \sigma _\beta {\mathscr {P}}(\varvec{D})\right] ^{-1} \, \exp \left( - \, \frac{1}{2}\sum _{j=1}^n\left( \frac{S_{(i+1)j}-D_j}{\gamma _j}\right) ^2\right. \nonumber \\&\left. - \,\frac{1}{2}\sum _{\beta =1}^\alpha \left( \frac{\theta _{(i+1)\beta }-\theta _{i\beta }}{\sigma _\beta }\right) ^2 \right) \ . \end{aligned}$$

**Step 2:**

The likelihood ratio is now calculated to be ${\mathscr {P}}(\varvec{\Theta _{i+1}|D}) / {\mathscr {P}}(\varvec{\Theta _i|D})$.

**Step 3:**

Next, we generate a uniform random number $r \sim U[0,1]$. If $r < {\mathscr {P}}(\varvec{\Theta _{i+1}|D})/{\mathscr {P}}(\varvec{\Theta _i|D})$, we accept $\varvec{\Theta _{i+1}}$, otherwise we go back to Step 1 and repeat the procedure.

We have used cumulative infected and dead data as the vector $\varvec{D}$ and we normalize (as described above) the data vector $\varvec{D}$, as well as the simulated vector $\varvec{S_i}$ at every step, before calculating the likelihood ratio in Step 2 above. We have used $\sigma = (\varvec{\sigma _P}, \varvec{\sigma _{IC}})$, where $\varvec{\sigma _P} = (0.01, 0.01, 0.01, 0.01, 0.01, 0.01, 0.01, 0.01, 0.01, 0.01, 0.01)$ only for parameters part, $\varvec{\sigma _{IC}} = (0.1, 0.1, 0.001, 0.0, 0.0, 0.0)$ for initial data part, and $\varvec{\Gamma }=(\gamma _1, \gamma _2, \ldots , \gamma _n)$, where $\gamma _j = (0.1-0.05)(j-1)/(n-1)+0.05$. The initial days (where the numbers are low) in the data are given relatively smaller weightage than the later days for fitting, as the noise level is higher initially, than the signal.

### Estimation of the reproduction number kinetics

Understandably, the basic reproduction number $R_0$ is no longer a constant. Defining $R_0(t)$ as the average number of secondary infections from a primary case at a given epoch *t*, and similarly $I_d(t)$ as the number of daily new cases, we have

7 $$\begin{aligned} I_d(t)= & {} \int _0^{\infty } R_0(t) \, I_d(t-\tau ) \, g(\tau ) \, d\tau , \end{aligned}$$

where $g(\tau )$ is the probability density function of the generation time $\tau$, defined as the time required for a new secondary infection to be generated from a primary infection. In other words, $\tau$ is the time interval between the onset of a primary case to the onset of a secondary case, generated from this primary case. As is reported^37^, the mean generation time is approximately 6.5 days, we assume $g(\tau )$ has a Gamma distribution with $g(\tau ) = \mathrm {Gamma}(6.5, 0.62)$. We represent $R_0(t)$ as a function of time as

8 $$\begin{aligned} R_0(t)= & {} \frac{I_d(t)}{\int _0^{\infty } I_d(t-\tau ) \, g(\tau ) \, d\tau }. \end{aligned}$$

We approximate the denominator of Eq. (8) directly from our simulated data, by a discrete sum, and evaluate $R_0$ at *n*th day as

9 $$\begin{aligned} R_0(n)= & {} \frac{I_d(t)}{\int _0^{\infty } I_d(t-\tau ) \, g(\tau ) \, d\tau } \approx \frac{I_d(n)}{\displaystyle \sum \nolimits _{\tau =0}^{n-1}I_d(n-\tau ) \, g(\tau )}. \end{aligned}$$

### Statistical error estimation and *p*-values

Using the Chi-square statistic as $\chi ^{2} \equiv \sum \nolimits _{i=1}^n \left( \frac{D_{i}-S_{i}}{\epsilon S_{i}+1}\right) ^{2}$ ($0<\epsilon <1$), where $D_i$ are observed data and $S_i$ the simulated data for the $\text {i}$th day, we quantify the accuracy of our model fitting with the real data. Understandably, the data for daily new infections and daily new deaths are contaminated by noise, more severely than the corresponding cumulative data. Hence, a Chi-square test applied on cumulative data will always give a high *p*-value. However, to test the power of our predictive machine learning algorithm, we calculated the *p*-values on daily new data of deaths and infected. Assuming the real data are drawn from a normal distribution with mean value same as the simulated data, and with a standard deviation equal to some fraction of the simulated data, we derive our Chi-square statistic. Although, the real data of infected and dead are always positive, as the infection increases, this assumption is very well valid, except for a very small time interval at the starting of infection in a population.

## Supplementary Information

Supplementary Information.

## Data Availability

Data from the Johns Hopkins repository (https://github.com/CSSEGISandData/Covid-19) were used, together with country specific repositories, e.g. US: https://usafacts.org; EU: https://data.europa.eu/; UK: https://coronavirus.data.gov.uk/; India: https://www.covid19india.org/. All the epidemiological information we used is documented in the Extended Data and Supplementary Tables. The codes and relevant files are made available through the Aston Data Repository.
