# Supplementary material for: Infection kinetics of Covid-19 and containment strategy
Source: Sci Rep. 2021 Jun 2;11:11606. doi: 10.1038/s41598-021-90698-2 (PMC8173017; doi:10.1038/s41598-021-90698-2)
Supplement: Supplementary file 1 — Supplementary Information. [file 41598_2021_90698_MOESM1_ESM.pdf]

Infection Kinetics of Covid-19 and Containment Strategy
ONLINE SUPPLEMENTARY

Amit K Chattopadhyay, Debajyoti Choudhury, Goutam Ghosh, Bidisha Kundu and Sujit Kumar Nath

Data training: In order to establish the predictive strength of this machine Learning enforced model, in the appendices, we trained the data between 10 February to 10 May and predicted for the next 30 days (until 9 June 2020). This is uniformly done for all countries.

Appendix I: Reproduction Number Dynamics for Class A, B, C, D Countries

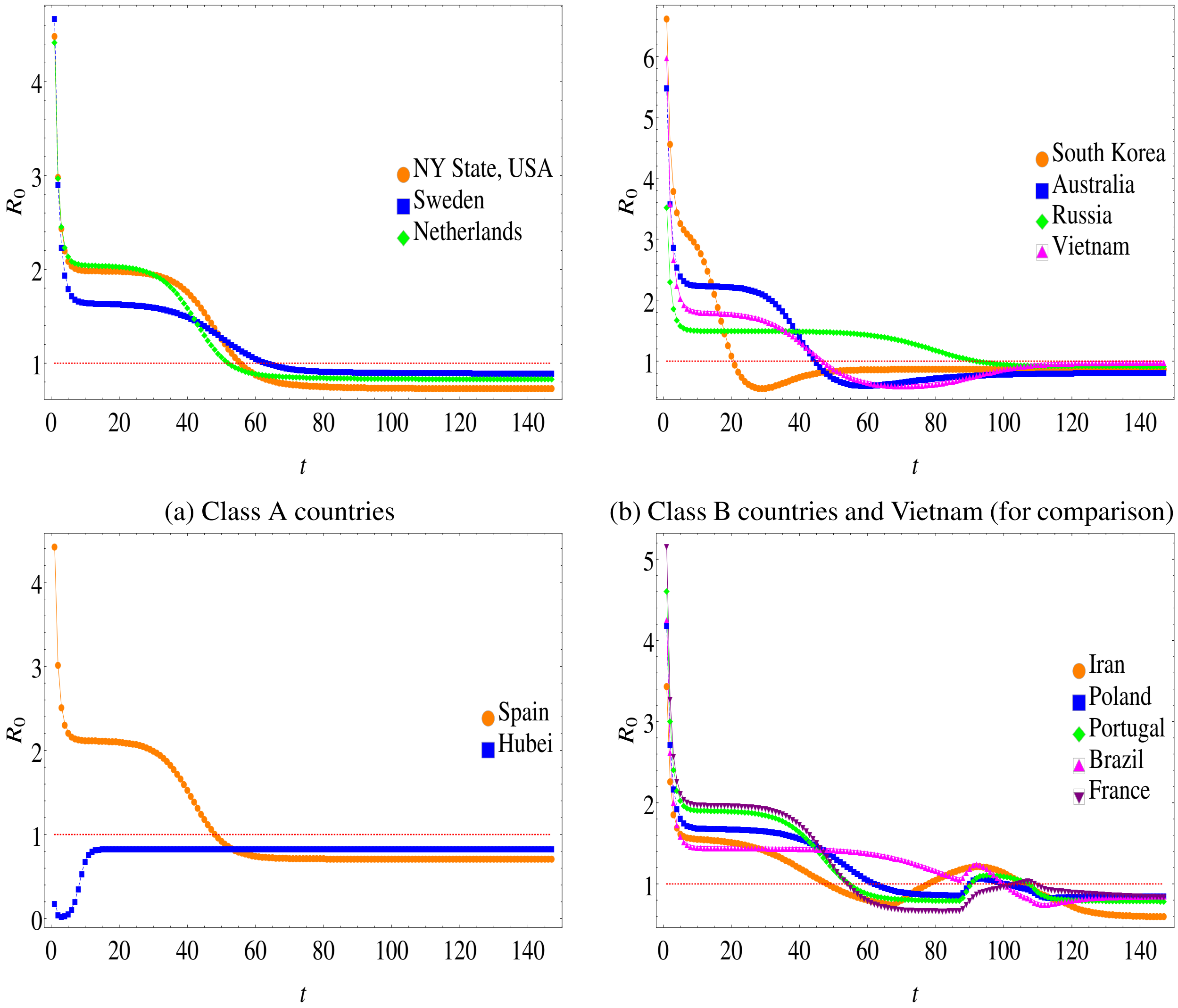

(a) Class A countries

(b) Class B countries and Vietnam (for comparison)

(c) Class C countries

(d) Class D countries

Figure S1: Temporal evolution of the basic reproduction rate for all 4 infection classes, on a day by day basis. The dotted line at $R_0 = 1$ points to the optimum above which the epidemic to pandemic regime starts.

Country	Daily New Infected		Daily New Death	
	ϵ	p-value	ϵ	p-value
Australia	0.48	0.31	0.12	0.18
Korea	0.6	0.48	0.3	0.19
NY state, USA	0.55	0.24	0.45	0.17
Poland	0.45	0.67	0.25	0.28
Russia	0.55	0.14	0.25	0.19
Belgium	0.55	0.46	0.35	0.26
Brazil	0.6	0.37	0.45	0.46
Hubei	1	0	1	0
Portugal	0.5	0.17	0.19	0.69
Spain	0.75	0.19	0.5	0.87
Sweden	0.6	0.28	0.5	0.2
Vietnam	0.9	0	0.1	1
Netherlands	0.4	0.63	0.31	0.41
Iran	0.45	0.21	0.35	0.37

Table S1: p-Values for daily new infected and dead for other Class A-D countries between 10 Feb to 10 May 2020. The statistic $\chi^2_D \equiv \sum_{i=1}^{n} \left(\frac{D_i - S_i}{\epsilon S_i + 1} \right)^2$ $(0 < \epsilon < 1)$ represents the chi-square value, where D_i are observed data and S_i the simulation data for the ith day.

Appendix II: Infection and mortality plots for countries in Classes A, B, C and D

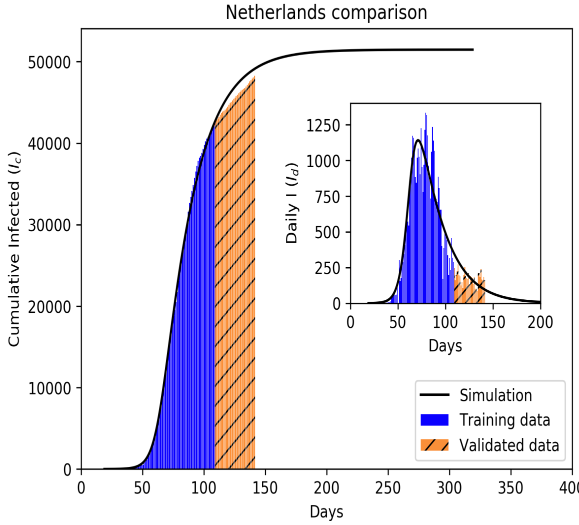

(a) Netherlands infection profiles.

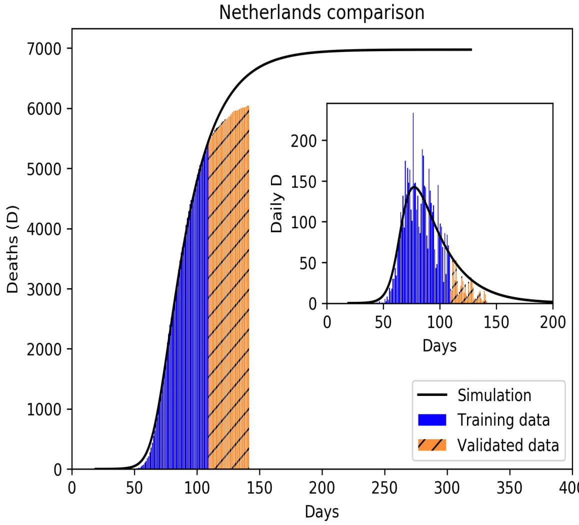

(b) Netherlands mortality profiles.

Figure S2: Infection (S2a) and mortality (S2b) epidemiology for Netherlands (Class A). The outsets all represent the cumulative statistics while the insets are for daily updates in the number of infected and death respectively.

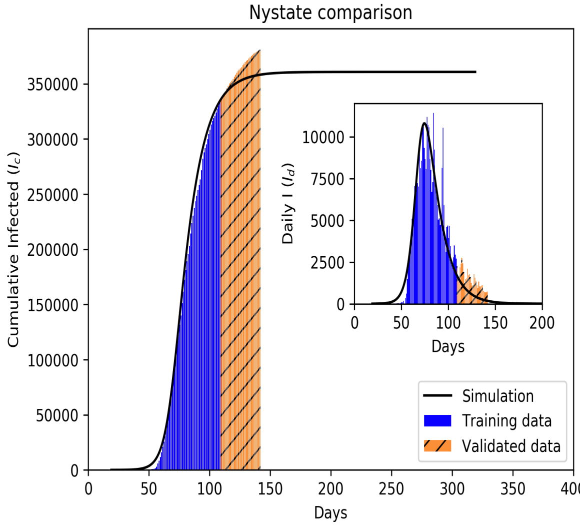

(a) New York State infection profiles.

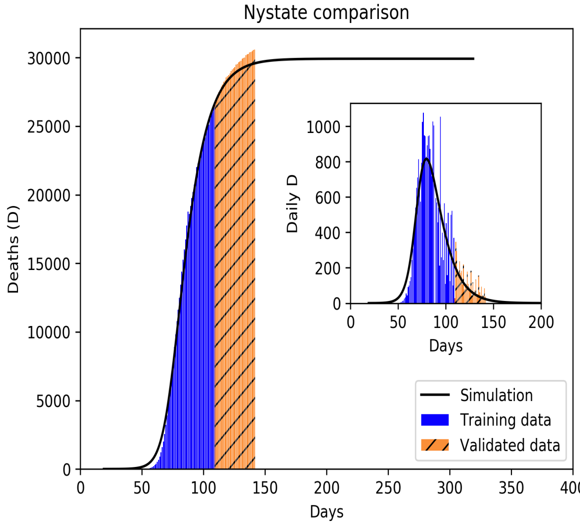

(b) New York State mortality profiles.

Figure S3: Infection (S3a) and mortality (S3b) epidemiology for New York State (Class A). The outsets all represent the cumulative statistics while the insets are for daily updates in the number of infected and death respectively.

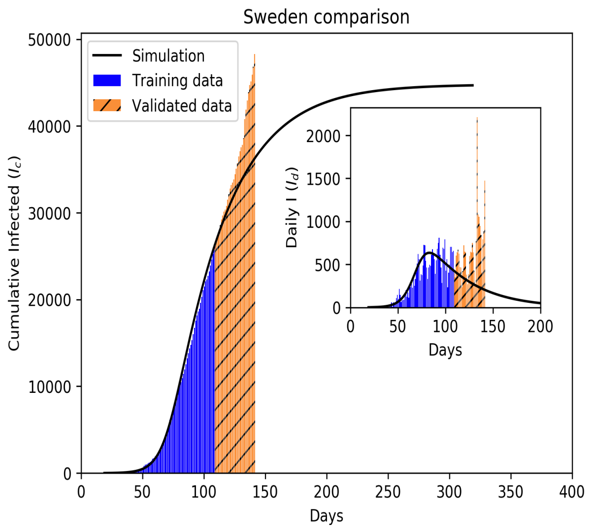

(a) Sweden infection profiles.

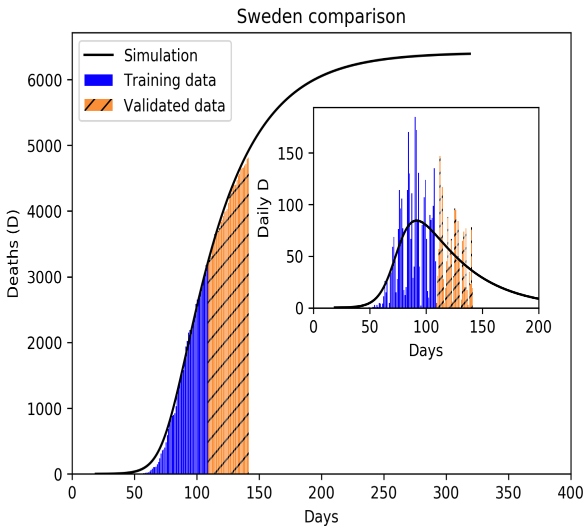

(b) Sweden mortality profiles.

Figure S4: Infection (S4a) and mortality (S4b) epidemiology for Sweden (Class A). The outsets all represent the cumulative statistics while the insets are for daily updates in the number of infected and death respectively.

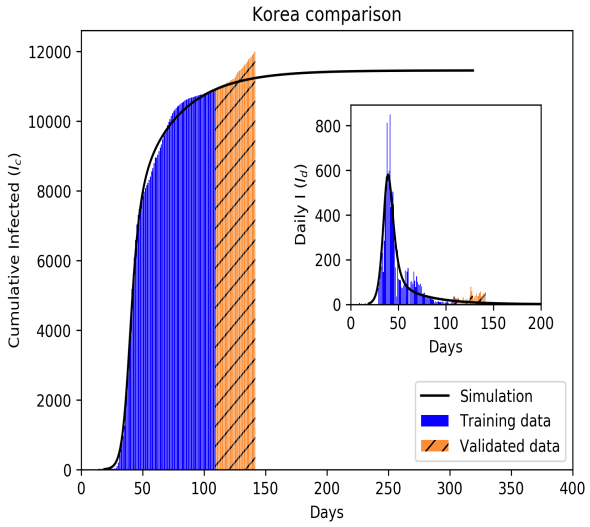

(a) Korea infection profiles.

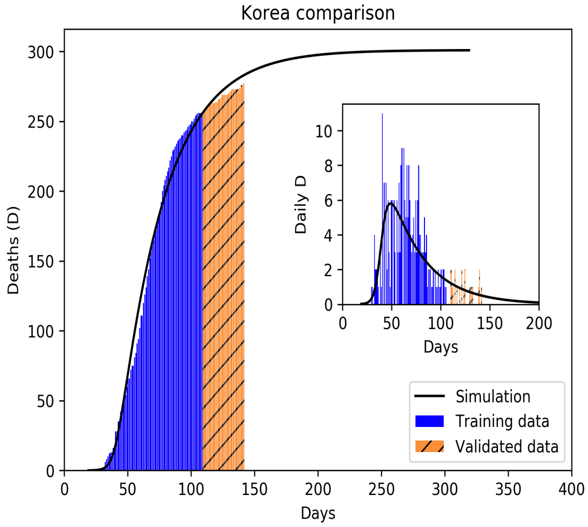

(b) Korea mortality profiles.

Figure S5: Infection (S5a) and mortality (S5b) epidemiology for Korea (Class B). The outsets all represent the cumulative statistics while the insets are for daily updates in the number of infected and death respectively.

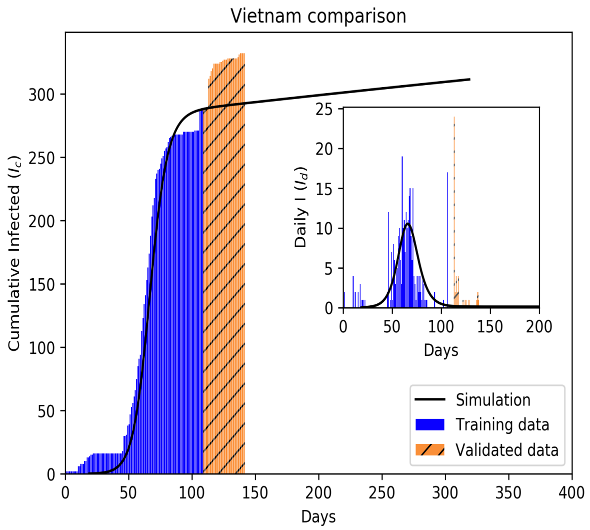

(a) Vietnam infection profiles.

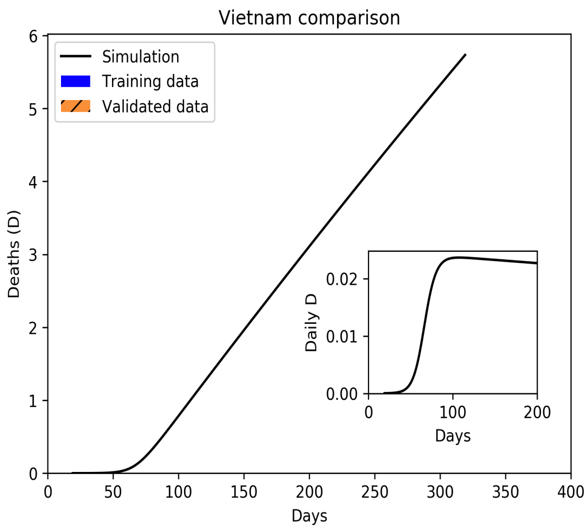

(b) Vietnam mortality profiles.

Figure S6: Infection (S6a) and mortality (S6b) epidemiology for Vietnam. The outsets all represent the cumulative statistics while the insets are for daily updates in the number of infected and death respectively.

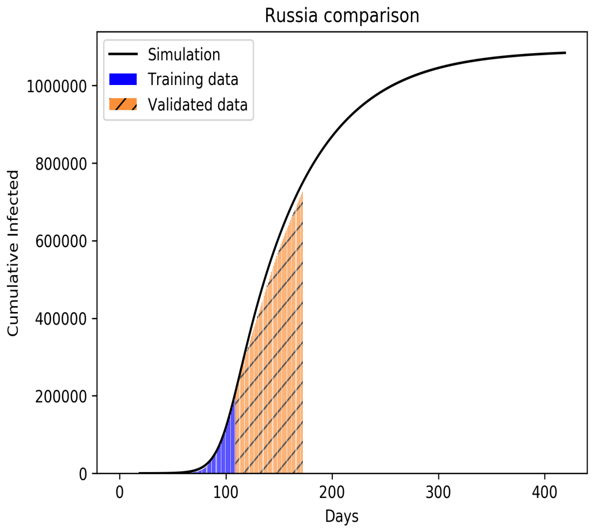

(a) Russia infection profiles.

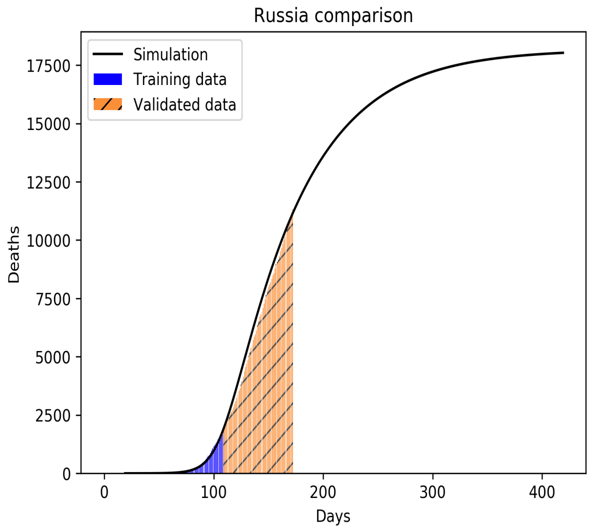

(b) Russia mortality profiles.

Figure S7: Infection (S7a) and mortality (S7b) epidemiology for Russia (Class B). The outsets all represent the cumulative statistics while the insets are for daily updates in the number of infected and death respectively.

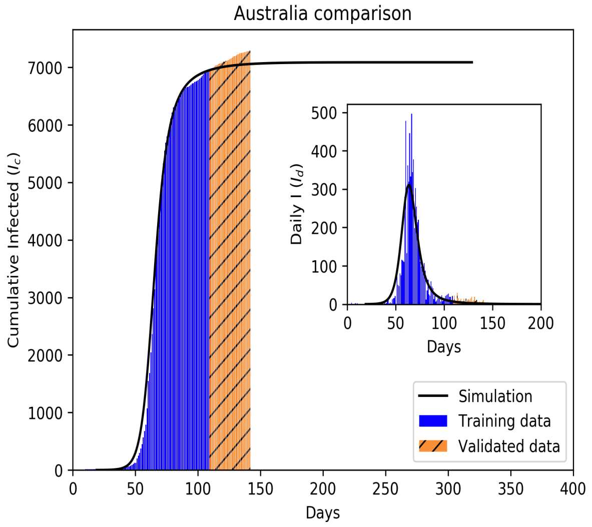

(a) Australia infection profiles.

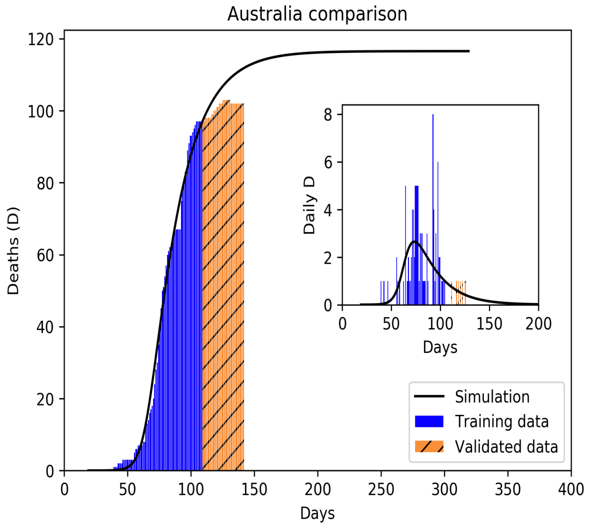

(b) Australia mortality profiles.

Figure S8: Infection (S8a) and mortality (S8b) epidemiology for Australia (Class B). The outsets all represent the cumulative statistics while the insets are for daily updates in the number of infected and death respectively.

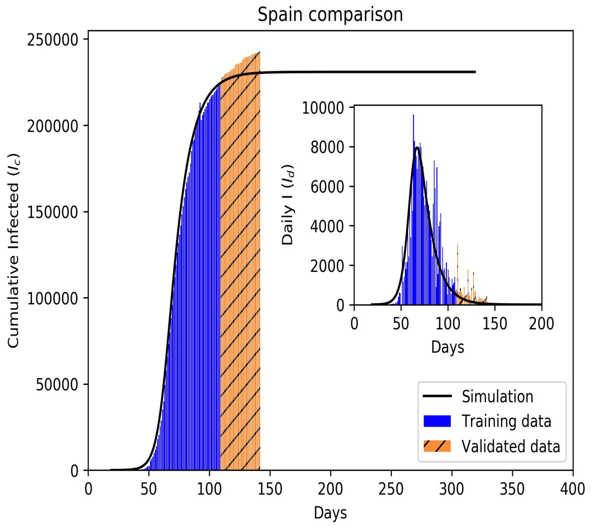

(a) Spain infection profiles.

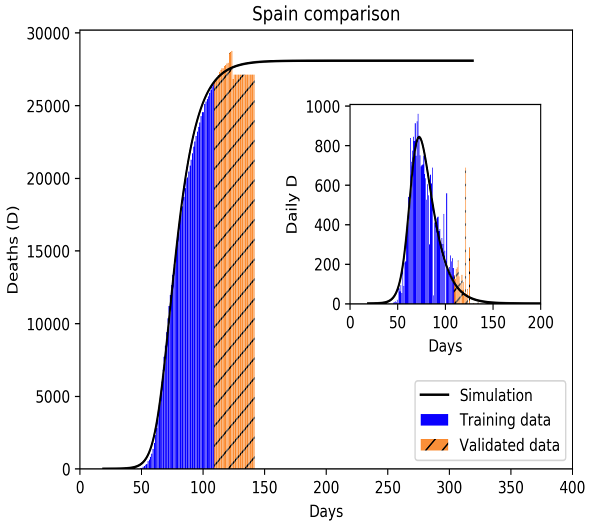

(b) Spain mortality profiles.

Figure S9: Infection (S9a) and mortality (S9b) epidemiology for Spain (Class C). The outsets all represent the cumulative statistics while the insets are for daily updates in the number of infected and death respectively.

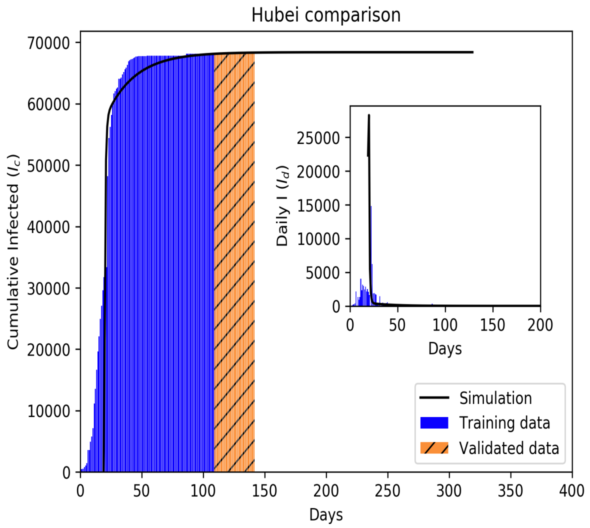

(a) Hubei infection profiles.

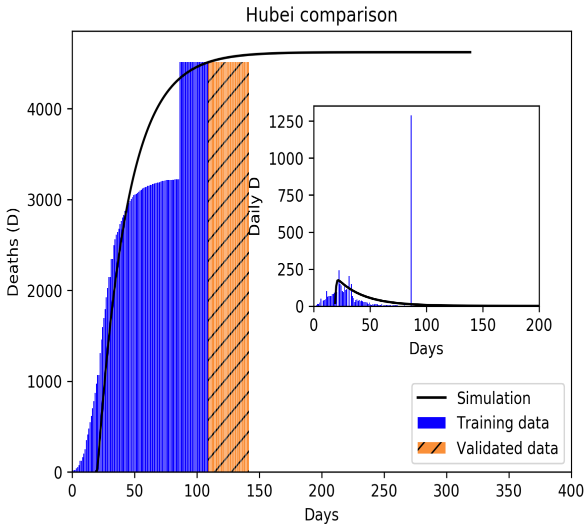

(b) Hubei mortality profiles.

Figure S10: Infection (S10a) and mortality (S10b) epidemiology for Hubei (Class C). The outsets all represent the cumulative statistics while the insets are for daily updates in the number of infected and death respectively.

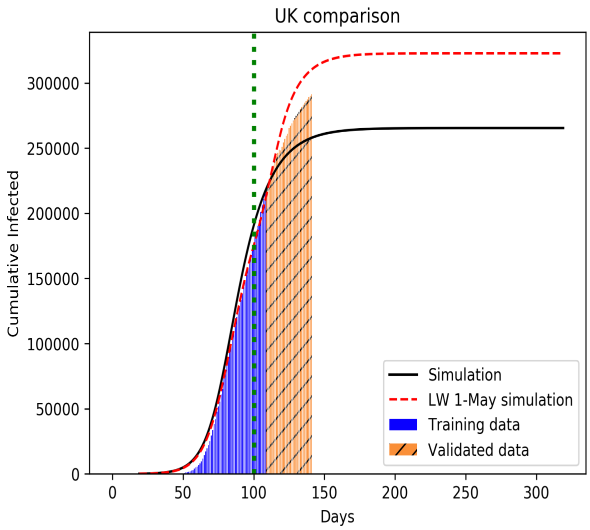

(a) UK: 1 May 2020

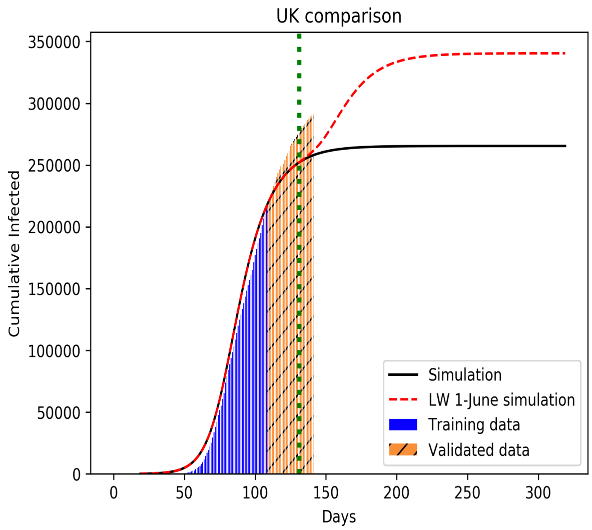

(b) UK: 1 June 2020

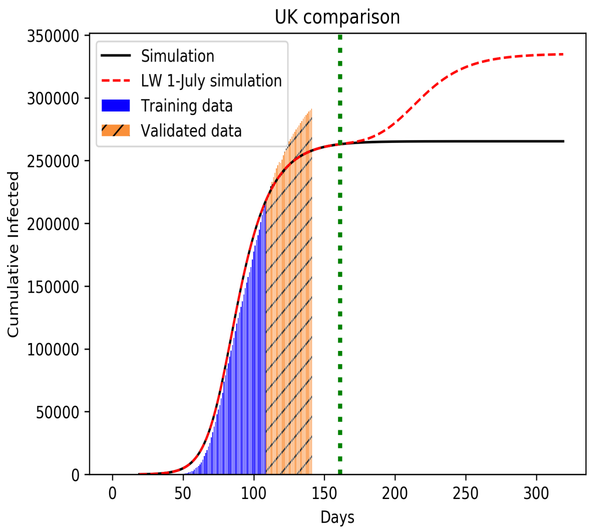

(c) UK: 1 July 2020

Figure S11: UK cumulative infected prediction for 3 different withdrawal dates - 1 May 2020, 1 June 2020, 1 July 2020.

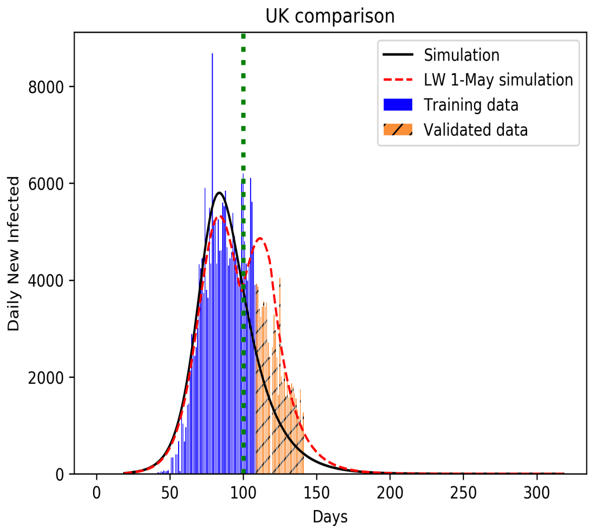

(a) UK: 1 May 2020

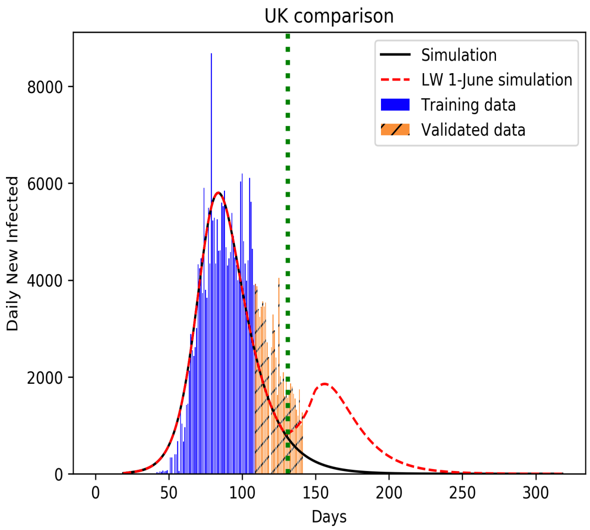

(b) UK: 1 June 2020

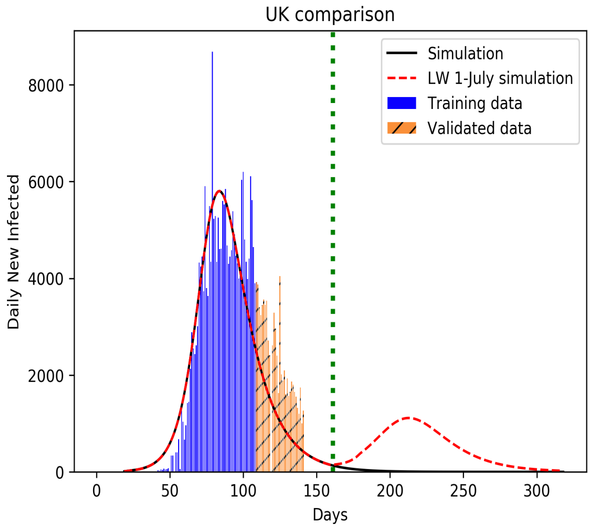

(c) UK: 1 July 2020

Figure S12: UK Lockdown prediction for 3 different withdrawal dates - 1 May 2020, 1 June 2020, 1 July 2020.

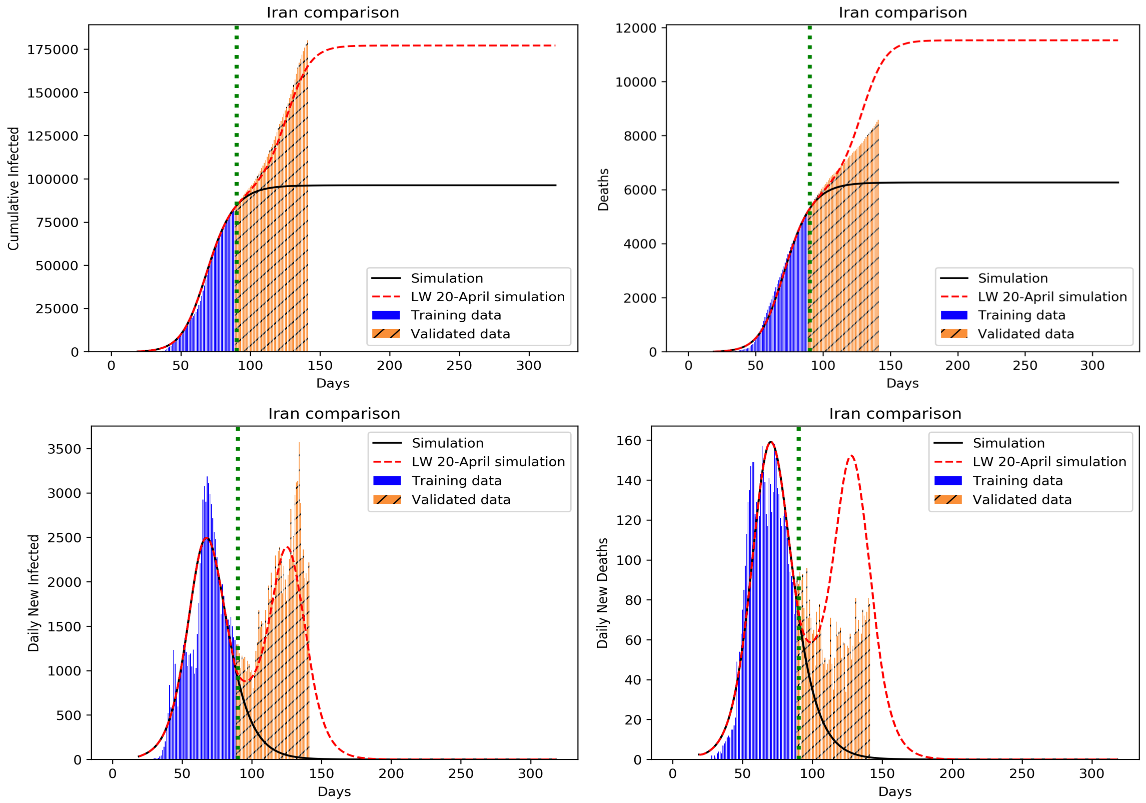

Figure S13: Iran cumulative infected and mortality prediction for lockdown withdrawal on 20 April 2020.

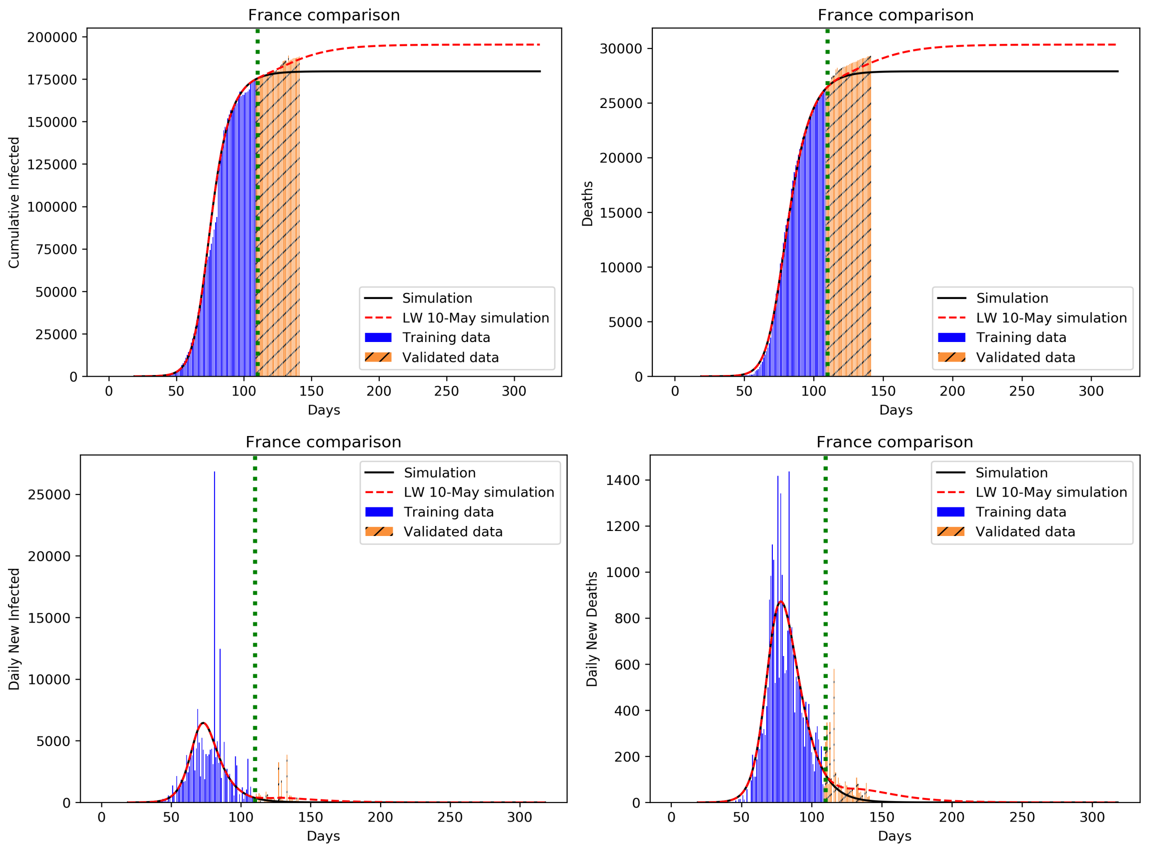

Figure S14: France cumulative infected and mortality prediction for lockdown withdrawal on 10 May 2020.

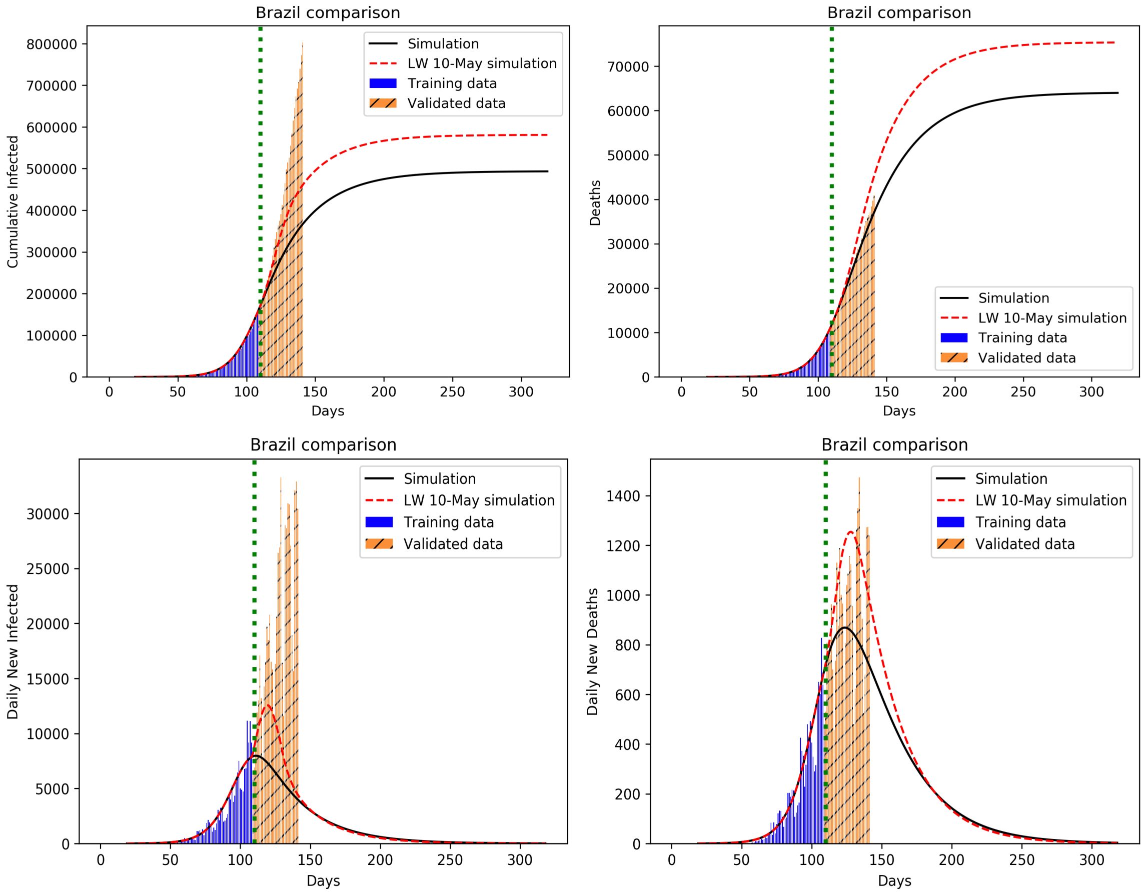

Figure S15: Brazil cumulative infected and mortality prediction for lockdown withdrawal on 10 May 2020.

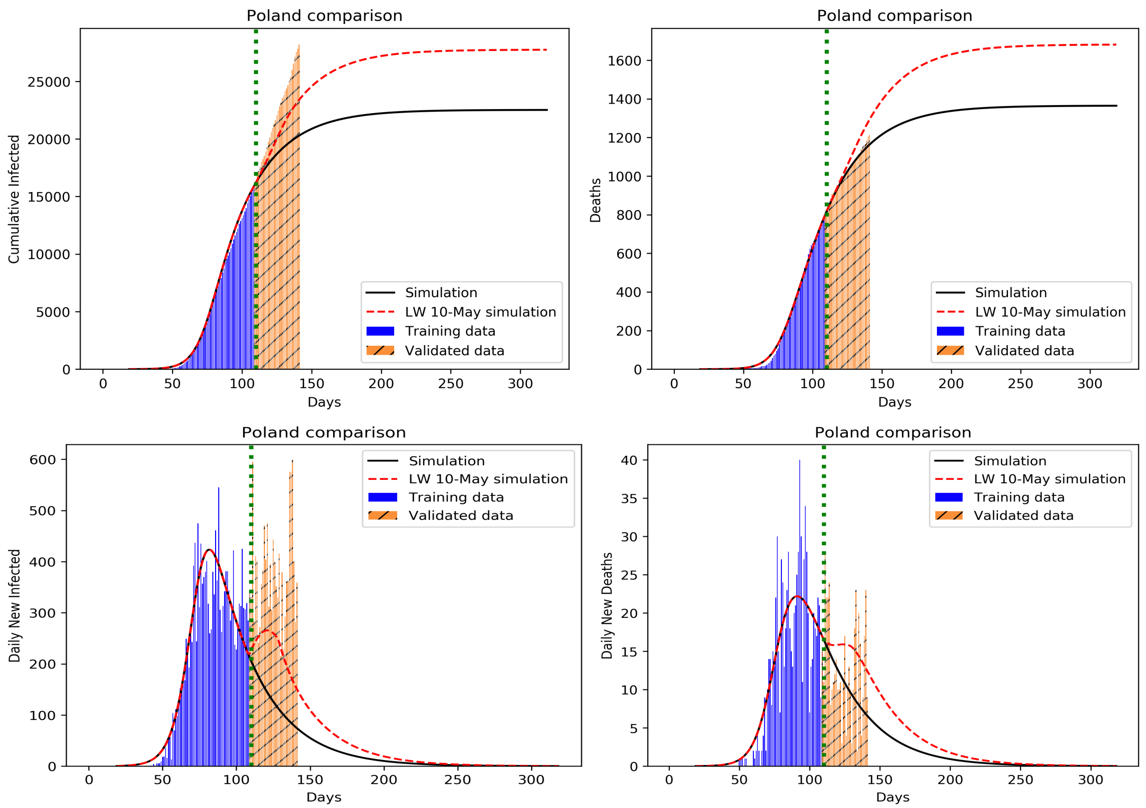

Figure S16: Poland cumulative infected and mortality prediction for lockdown withdrawal on 10 May 2020.

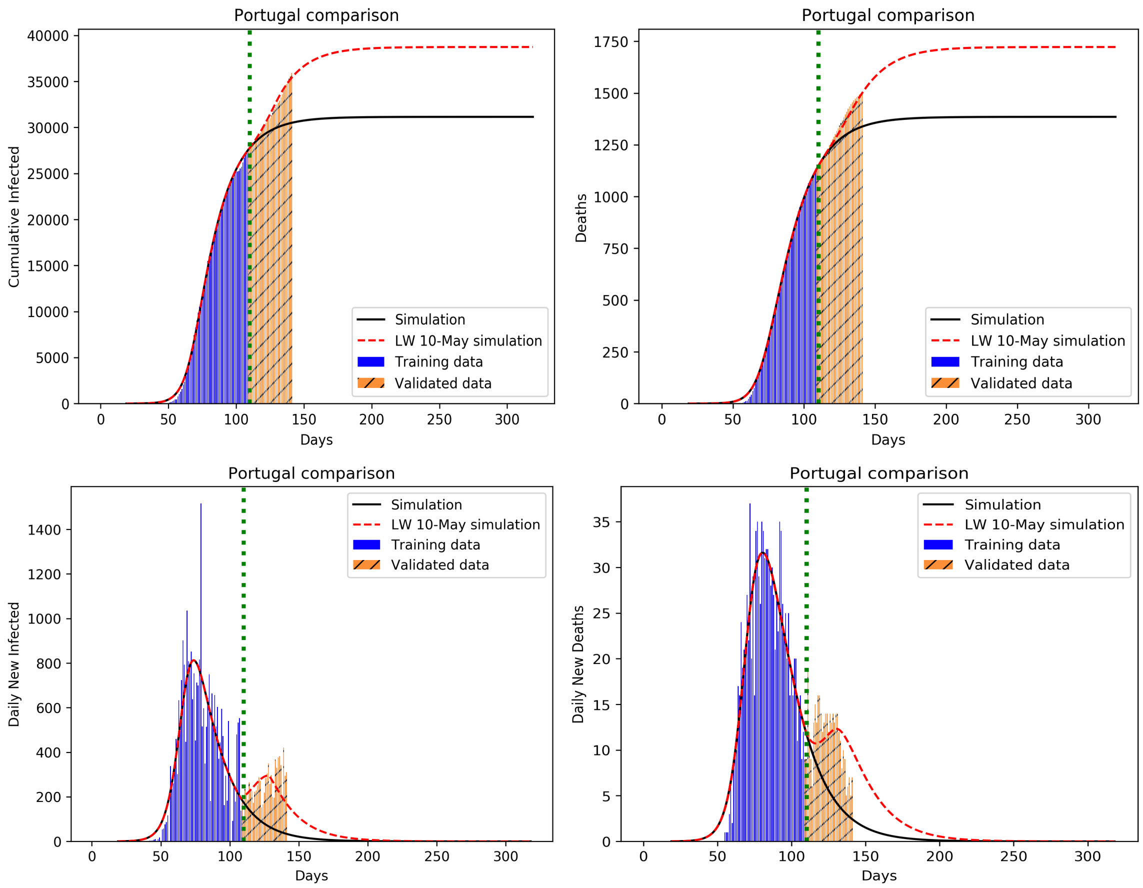

Figure S17: Portugal cumulative infected and mortality prediction for lockdown withdrawal on 10 May 2020.

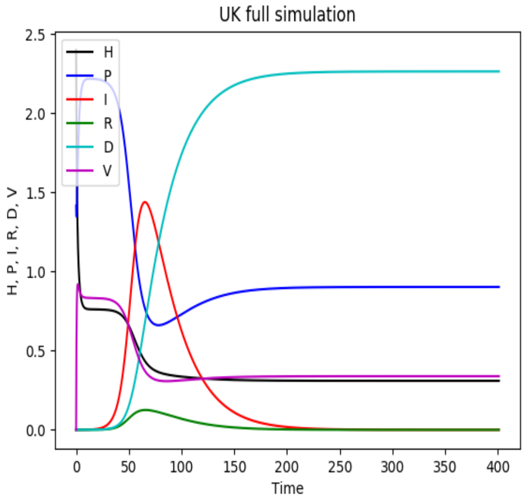

(a) Full simulation plots including all 6 dimensions for the UK

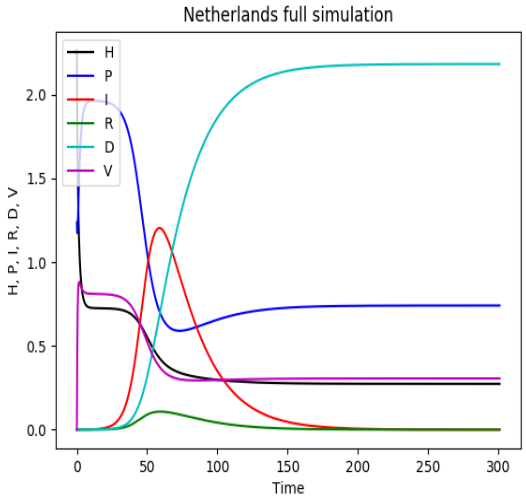

(b) Full simulation plots including all 6 dimensions for the Netherlands

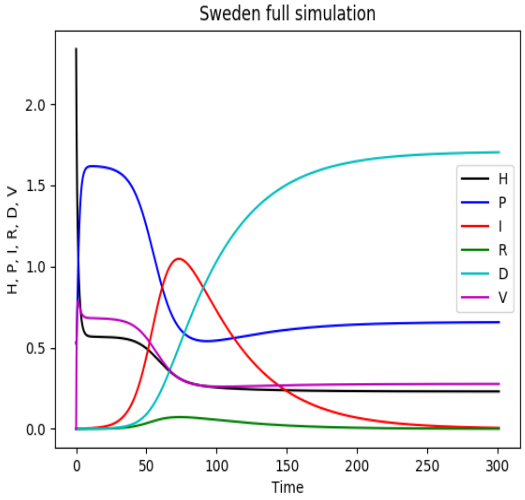

(c) Full simulation plots including all 6 dimensions for Sweden

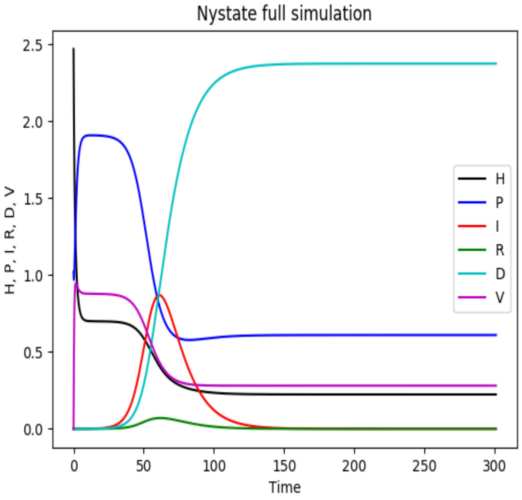

(d) Full simulation plots including all 6 dimensions for New York State

Figure S18: Full Simulation plots for Class A countries.

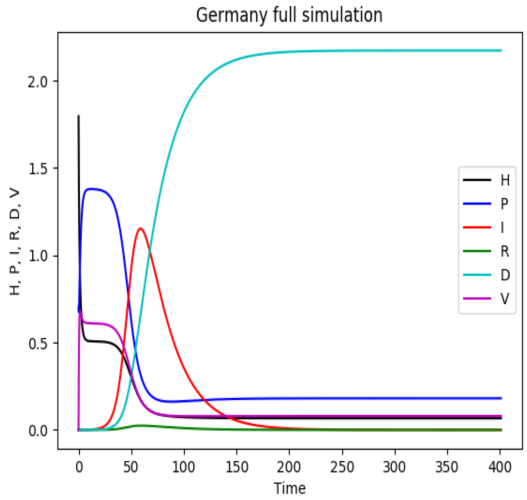

(a) Full simulation plots including all 6 dimensions for Germany

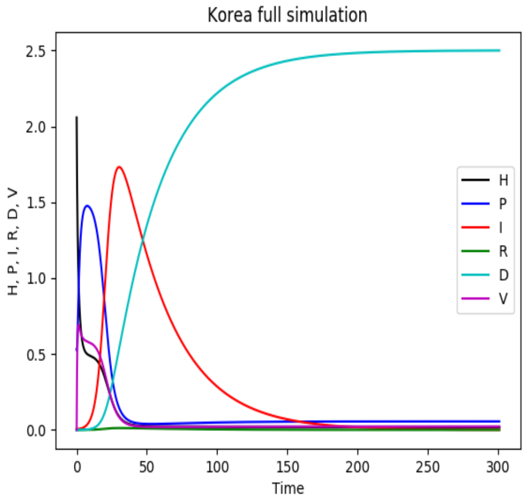

(b) Full simulation plots including all 6 dimensions for Korea

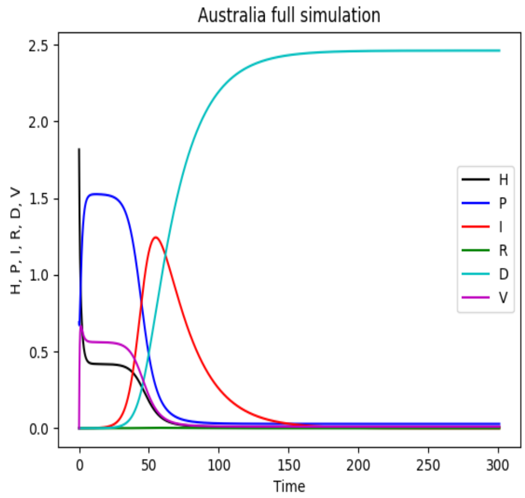

(c) Full simulation plots including all 6 dimensions for Australia

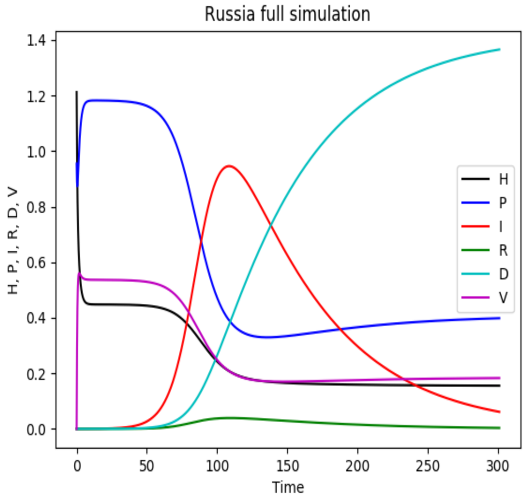

(d) Full simulation plots including all 6 dimensions for Russia

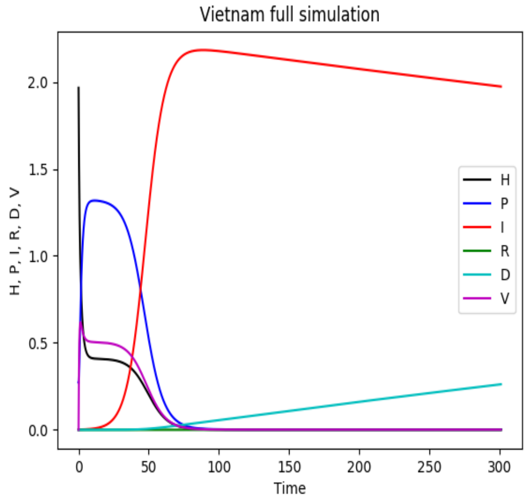

(e) Full simulation plots including all 6 dimensions for Vietnam

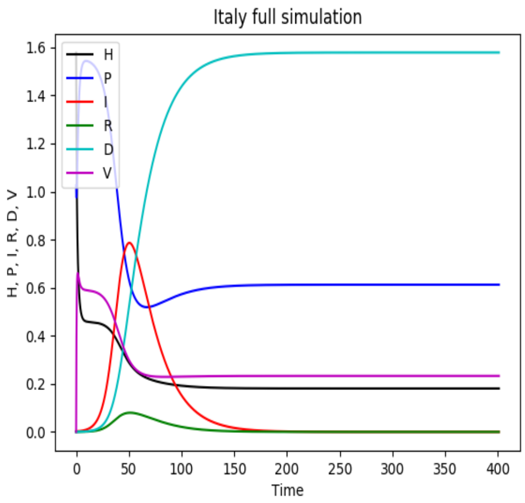

(a) Full simulation plots including all 6 dimensions for Italy

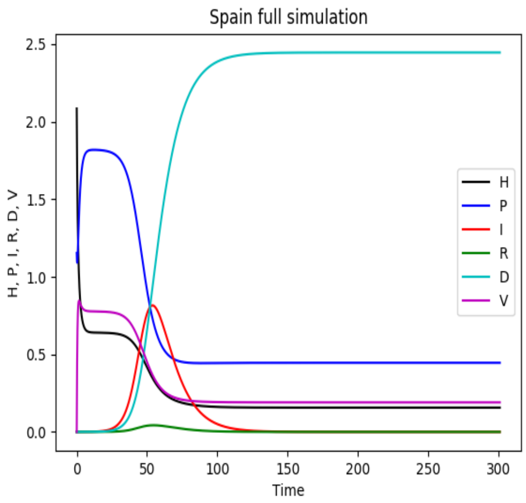

(b) Full simulation plots including all 6 dimensions for Spain

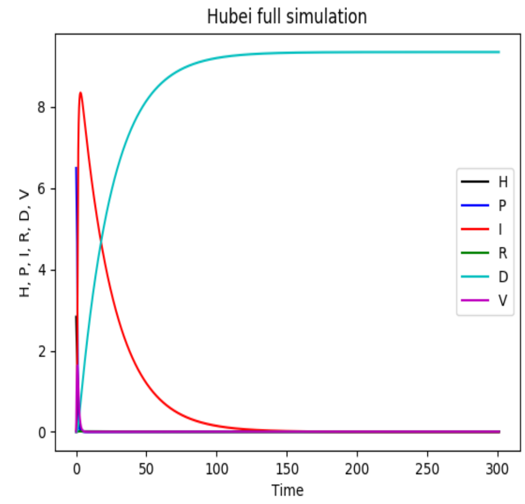

(c) Full simulation plots including all 6 dimensions for Hubei

Figure S20: Full Simulation plots for Class C countries.

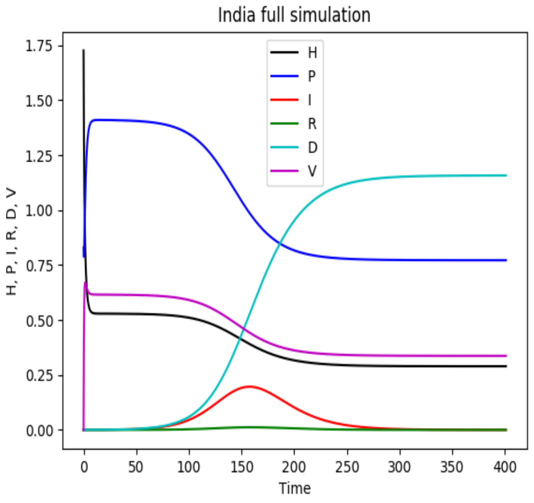

(a) Full simulation plots including all 6 dimensions for India

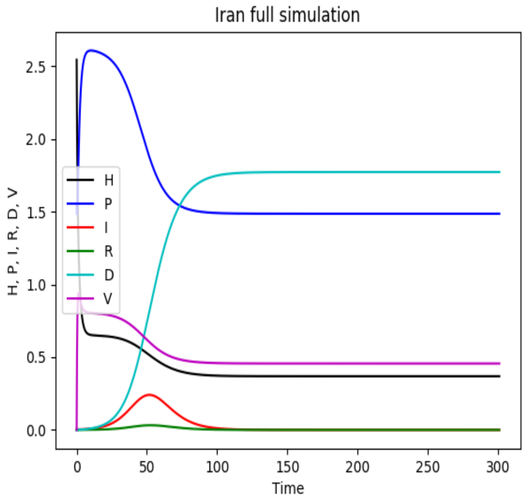

(b) Full simulation plots including all 6 dimensions for Iran

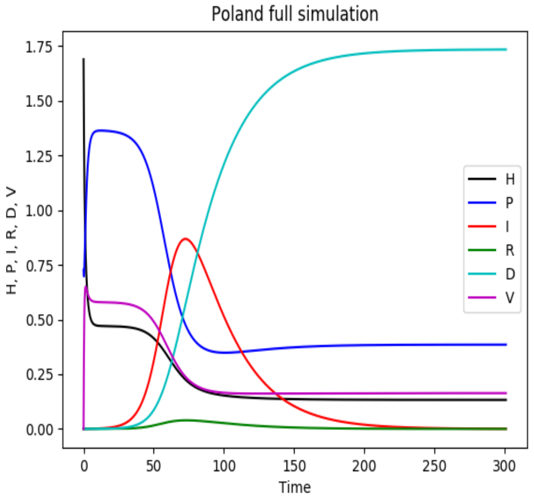

(c) Full simulation plots including all 6 dimensions for Australia

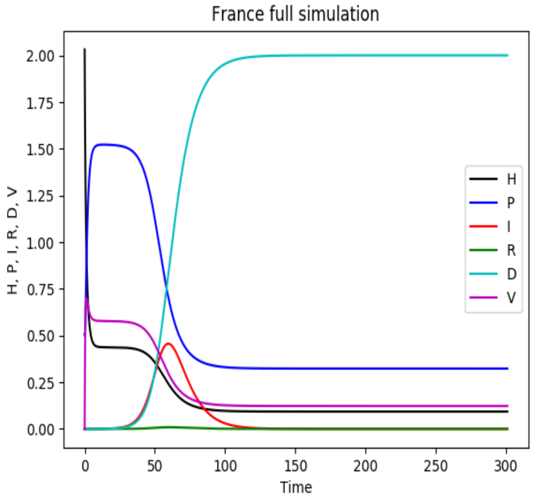

(d) Full simulation plots including all 6 dimensions for France

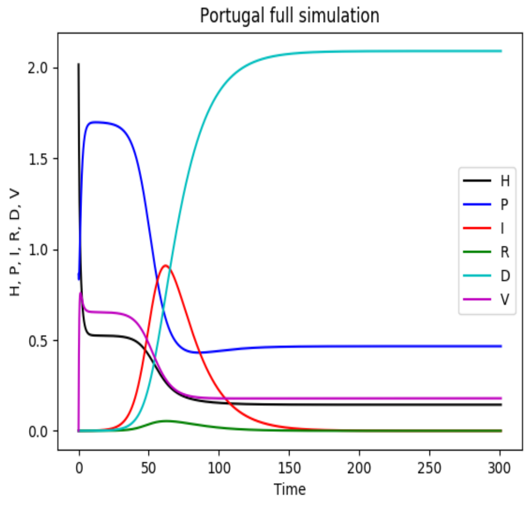

(e) Full simulation plots including all 6 dimensions for Portugal

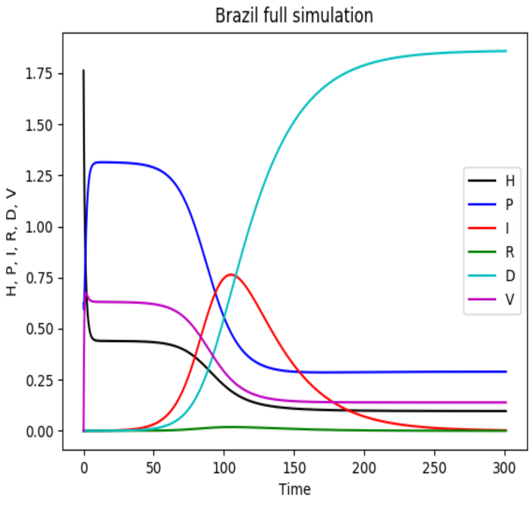

(f) Full simulation plots including all 6 dimensions for Brazil

Appendix III: Comparative Estimation: Data versus Model Prediction Table

Days \ Country	Netherlands				Sweden				New York State, USA				Belgium			
	Infected		Death		Infected		Death		Infected		Death		Infected		Death	
	Data	Simulation	Data	Simulation	Data	Simulation	Data	Simulation	Data	Simulation	Data	Simulation	Data	Simulation	Data	Simulation
31/05/20	102	151	5	26	775	266	65	45	1329	395	51	51	98	144	19	45
01/06/20	86	145	10	25	2214	260	74	44	1045	368	51	48	70	138	17	43
02/06/20	209	139	13	24	1080	254	20	43	1048	343	155	45	82	132	26	41
03/06/20	210	134	15	23	1056	248	77	42	1075	319	62	42	140	126	18	39
04/06/20	183	128	6	22	948	242	17	41	1108	297	44	39	165	120	14	37
05/06/20	239	123	2	21	843	237	3	41	781	277	94	36	154	115	15	36
06/06/20	165	118	3	20	403	231	35	40	702	258	43	34	122	110	11	34
07/06/20	164	113	15	19	791	226	23	39	683	240	41	31	89	105	13	33
08/06/20	184	109	11	19	890	221	78	38	674	224	84	29	132	100	10	31
09/06/20	164	105	2	18	1474	216	19	37	736	208	38	27	142	96	7	30

Table S2: Validation of Daily new Infected and Death: Netherlands, Sweden, New York State, Belgium (Class A)

Days \ Country	Korea				Australia				Russia			
	Infected		Death		Infected		Death		Infected		Death	
	Data	Simulation	Data	Simulation	Data	Simulation	Data	Simulation	Data	Simulation	Data	Simulation
31/05/20	49	8	1	0	8	2	0	0	8858	9766	182	168
01/06/20	39	8	0	0	11	2	0	0	8529	9632	177	167
02/06/20	39	8	0	0	7	2	0	0	8823	9497	168	167
03/06/20	51	7	0	0	5	1	0	0	8718	9364	144	1660
04/06/20	57	7	0	0	7	1	0	0	8846	9231	197	165
05/06/20	38	7	0	0	6	1	0	0	8971	9100	134	164
06/06/20	38	7	1	0	2	1	0	0	8970	8970	112	163
07/06/20	50	6	2	0	7	1	0	0	8587	8841	171	162
08/06/20	45	6	0	0	11	1	0	0	8393	8714	216	161
09/06/20	56	6	1	0	4	1	0	0	8777	8588	172	159

Table S3: Validation of Daily new Infected and Death: Korea, Australia, Russia (Class B)

Days \ Country	Spain				Hubei Province, China			
	Infected		Death		Infected		Death	
	Data	Simulation	Data	Simulation	Data	Simulation	Data	Simulation
31/05/20	294	95	0	21	0	4	0	1
01/06/20	394	88	1	20	0	4	0	1
02/06/20	334	82	5	18	0	4	0	1
03/06/20	318	75	1	17	0	3	0	1
04/06/20	332	70	1	15	0	3	0	1
05/06/20	240	64	1	14	0	3	0	1
06/06/20	167	60	0	13	0	3	0	1
07/06/20	249	55	0	12	0	3	0	1
08/06/20	314	51	0	11	0	3	0	1
09/06/20	427	47	0	10	0	3	0	1

Table S4: Validation of Daily new Infected and Death: Spain, Hubei (Class C)

Country / Days	Poland				Iran				Francs				Portugal				Brazil			
	Infected		Death		Infected		Death		Infected		Death		Infected		Death		Infected		Death	
	Data	Simulation	Data	Simulation	Data	Simulation	Data	Simulation	Data	Simulation	Data	Simulation	Data	Simulation	Data	Simulation	Data	Simulation	Data	Simulation
31/05/20	230	231	18	15	3117	2222	64	150	0	375	107	59	195	279	12	12	28936	8346	1262	1242
01/06/20	292	222	23	15	3134	2152	70	147	3856	370	81	59	366	269	11	12	28633	7777	1349	1229
02/06/20	361	213	2	15	3574	2073	59	144	0	365	43	59	331	258	8	12	30925	7269	1473	1212
03/06/20	362	204	20	14	2886	1987	63	140	552	359	46	58	377	247	10	12	30830	6815	1005	1192
04/06/20	576	196	16	14	2269	1896	75	136	529	354	31	58	382	237	9	11	27075	6409	904	1170
05/06/20	575	189	4	14	2364	1803	72	131	293	347	13	57	342	226	5	11	18912	6043	525	1146
06/06/20	599	181	9	14	2043	1707	70	126	98	341	53	56	192	216	6	11	15654	5712	679	1120
07/06/20	400	175	17	13	2095	1612	74	120	141	334	84	56	421	206	7	11	32091	5413	1272	1094
08/06/20	282	168	23	13	2011	1517	81	115	397	327	23	55	294	196	5	10	32913	5141	1274	1066
09/06/20	359	162	9	13	2218	1415	78	109	358	320	27	54	310	187	7	10	30412	4892	1239	1039

Table S5: Validation of Daily new Infected and Death: Poland, Iran, France, Portugal, Brazil (Class D)
